# MFN2 Deficiency Impairs Mitochondrial Functions and PPAR Pathway During Spermatogenesis and Meiosis in Mice

**DOI:** 10.3389/fcell.2022.862506

**Published:** 2022-04-14

**Authors:** Tianren Wang, Yuan Xiao, Zhe Hu, Jingkai Gu, Renwu Hua, Zhuo Hai, Xueli Chen, Jian V. Zhang, Zhiying Yu, Ting Wu, William S. B. Yeung, Kui Liu, Chenxi Guo

**Affiliations:** ^1^ Shenzhen Key Laboratory of Fertility Regulation, Reproductive Medicine Center, The University of Hong Kong-Shenzhen Hospital, Shenzhen, China; ^2^ Department of Obstetrics and Gynaecology, Li Ka Shing Faculty of Medicine, The University of Hong Kong, Hong Kong SAR, China; ^3^ Center for Energy Metabolism and Reproduction, Shenzhen Institute of Advanced Technology, Chinese Academy of Sciences, Shenzhen, China; ^4^ Shenzhen Institute of Advanced Technology, Chinese Academy of Sciences, Shenzhen, China; ^5^ Department of Gynecology, Shenzhen Second People’s Hospital/The First Affiliated Hospital of Shenzhen University Health Science Center, Shenzhen, China; ^6^ Department of Gynecology and Obstetrics, The University of Hong Kong-Shenzhen Hospital, Shenzhen, China

**Keywords:** spermatogenesis, meiosis, mitofusin 2, lipid metabolism, mitochondrial dynamics

## Abstract

Mitochondria are highly dynamic organelles and their activity is known to be regulated by changes in morphology via fusion and fission events. However, the role of mitochondrial dynamics on cellular differentiation remains largely unknown. Here, we explored the molecular mechanism of mitochondrial fusion during spermatogenesis by generating an *Mfn2* (mitofusin 2) conditional knock-out (cKO) mouse model. We found that depletion of MFN2 in male germ cells led to disrupted spermatogenesis and meiosis during which the majority of Mfn2 cKO spermatocytes did not develop to the pachytene stage. We showed that in these Mfn2 cKO spermatocytes, oxidative phosphorylation in the mitochondria was affected. In addition, RNA-Seq analysis showed that there was a significantly altered transcriptome profile in the *Mfn2* deficient pachytene (or pachytene-like) spermatocytes, with a total of 262 genes up-regulated and 728 genes down-regulated, compared with wild-type (control) mice. Pathway enrichment analysis indicated that the peroxisome proliferator-activated receptor (PPAR) pathway was altered, and subsequent more detailed analysis showed that the expression of PPAR α and PPAR γ was up-regulated and down-regulated, respectively, in the MFN2 deficient pachytene (or pachytene-like) spermatocytes. We also demonstrated that there were more lipid droplets in the *Mfn2* cKO cells than in the control cells. In conclusion, our study demonstrates a novel finding that MFN2 deficiency negatively affects mitochondrial functions and alters PPAR pathway together with lipid metabolism during spermatogenesis and meiosis.

## Introduction

Mitochondria are known to play a critical role in controlling cell metabolism in eukaryotic cells, as they act as a power station to generate ATP via the oxidation of nutrients. At the same time, this organelle is highly active that can change its shape via fusion and fission events, and in this way to facilitate the metabolic milieu and other important cellular activities. These fusion and fission processes have long been recognized to regulate mitochondrial dynamics (Thorsness 1992; Bereiter-Hahn and Vöth 1994; Chan 2012; Mishra and Chan 2016). Indeed, the first time that mitochondria were shown to be a highly dynamic organelles was more than a century ago when in 1914 mitochondrial “granules” in chick embryonic cells were demonstrated to fuse together (Lewis and Lewis 1914). However, the molecular function of this mysterious behavior remained unknown for more than 80 years, until some of the molecular components that mediate these dynamic events were first identified in 2000 (Mozdy et al., 2000). One such component is mitofusin 2 (MFN2). This is a mitochondrial outer membrane GTPase, which was identified as playing a role in mitochondrial fusion and quality control. More recently, MFN2 has also been shown to play an essential role in regulating mitochondrial-endoplasmic reticulum (ER) tethering as well as cell metabolism in mammalian cells (Chen and Chan 2005; de Brito and Scorrano 2008; Filadi et al., 2018; Emery and Ortiz 2021).

In recent years, mitochondrial dynamics have been reported to play a crucial role during spermatogenesis and male fertility (Ren et al., 2019; Vertika et al., 2020). For example, the mitochondrial fission related factor, DRP1, was reported to play a role in maintaining mitochondrial function and spindle migration during meiosis in the oocytes of mice (Pan et al., 2020). Moreover, RAB7 was shown to regulate the phosphorylation of DRP1 and thus control mitochondrial dynamics during oocytogenesis (Pan et al., 2020). In addition to DRP1, several studies demonstrated that the conditional knock-out of mitofusin 1 or 2 (MFN1 or MFN2) leads to male or female infertility in mice models (Varuzhanyan et al., 2019; Zhang et al., 2019; Chen et al., 2020; Wang et al., 2021). However, the underlying molecular mechanism responsible for this phenotype remains largely unknown. Unlike some germ cell specific genes, which are known to directly participate in meiosis (Li et al., 2019; Li et al., 2021), MFN2 might play an indirect role in spermatogenesis by regulating various mitochondria related biological functions, such as oxidative phosphorylation and/or providing a supply ATP (Varuzhanyan et al., 2019). Although it is well-known that mitochondrial morphology plays a role in maintaining cellular homeostasis, there is limited knowledge about the role of morphological remodeling in germ cell development, and the underlying molecular mechanisms are also still unknown.

Here, based on previous studies on mitofusin proteins and mitochondrial dynamics, we have taken advantage of the Cre-LoxP system and generated a conditional knock-out (cKO) mouse line, which can specifically knock-out MFN2 in male postnatal germ cells. In this MFN2 cKO line, we analyzed the gross morphology and inner structure of the testis. We also investigated the role of MFN2 in spermatogenesis and meiosis of male germ cells. In addition, we isolated spermatocytes with high purity and performed RNA sequencing. We found that PPAR signaling was significantly affected by genetic deletion of the *Mfn2* gene. Furthermore, we also identified changes in lipid metabolism in MFN2 deficient meiotic cells. Overall, our data provide important insights into mitochondrial dynamics, and reveal a novel role of MFN2 in spermatogenesis via regulating the PPAR signaling pathway and lipid metabolism.

## Materials and Methods

### Mouse Maintenance

All the mice used in this study were of the C57BL/6J genetic background and they were generated by Caygen Biosciences (Santa Clara, California). The animals were housed under a controlled environment with free access to water and food, and with lights switched on between 6:00 and 18:00. All experimental protocols were approved by the regional ethics committee of the University of Hong Kong-Shenzhen Hospital.

### Generation of the *Mfn2* Conditional Knock-Out Mouse Line and Genotyping

**FIGURE 1:**
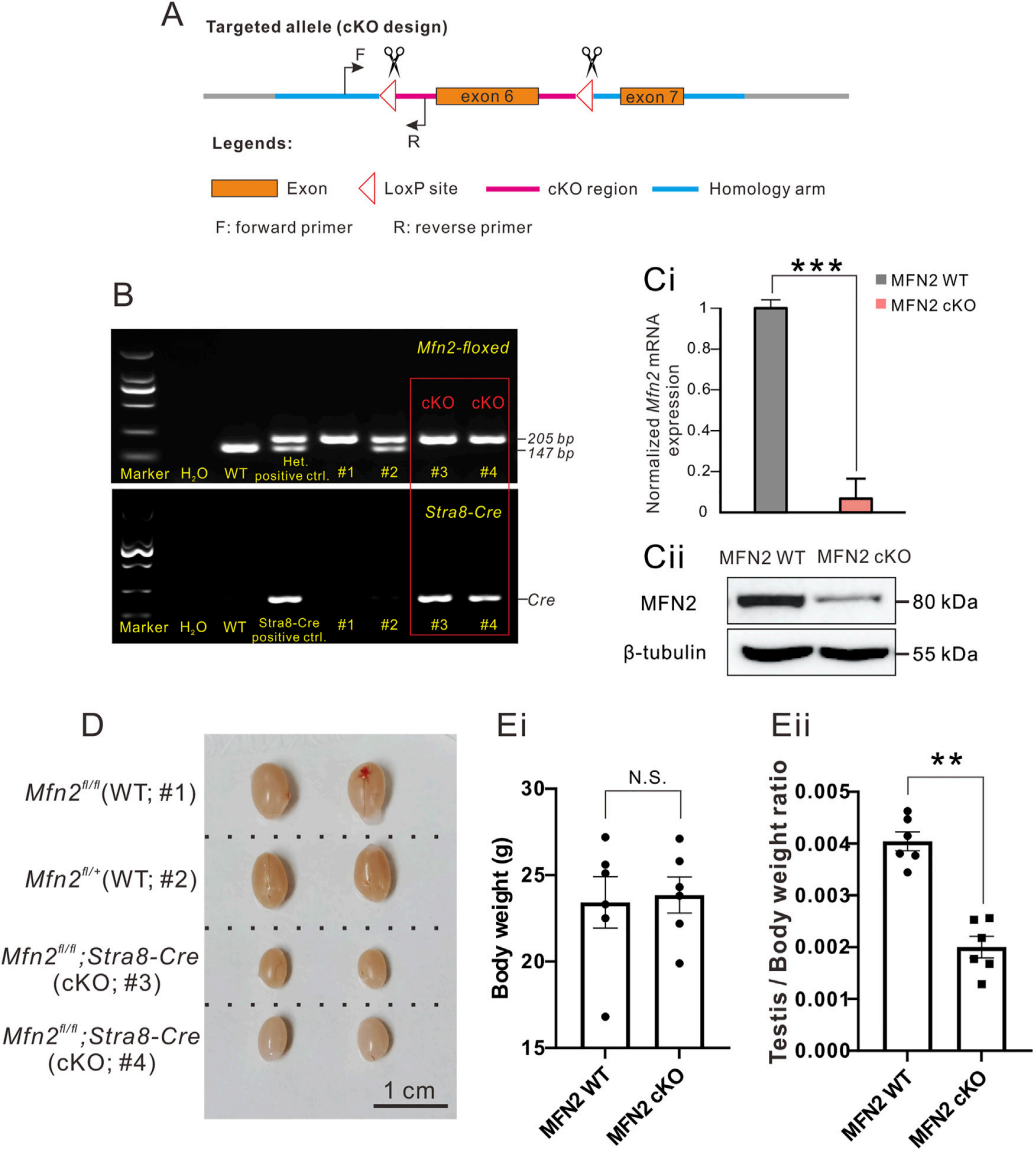
MFN2 conditional knock-out (cKO) mice demonstrate decreased testis size. **(A)** Design of the Cre-LoxP cKO system in the Mfn2 allele, such that exon 6 was flanked by two LoxP regions. When combined with Cre protein, exon 6 is deleted to complete Mfn2 gene knock-out. The forward and reverse primers were designed to check the insertion of the LoxP site in the Mfn2 allele. **(B)** Genotyping of the Mfn2;Stra8-Cre cKO mice. Tail DNA from wild-type (WT) mice was used as a negative control for PCR data analysis, whereas that from the Mfn2^fl/+^ heterozygous mouse was used as a positive control as there was a high band (205 base pairs, indicating successful insertion of LoxP in one of the Mfn2 alleles) and a low band (147 base pairs, indicating the Mfn2 wild-type allele, without LoxP insertion). Tail DNA from the Stra8-Cre mouse was used as a positive control to amplify a Cre band for PCR analysis. **(C)** Western blotting analysis to show the level of MFN2 level in the spermatocytes of MFN2 WT and MFN2 cKO mice. *ß*-tubulin was used as a loading control. **(D)** Representative examples of the testes isolated from two MFN2 WT and two MFN2 cKO mice, which were collected on postnatal day (PD) 56. The scale bar is 1 cm. **(E)** Bar charts to show the mean ± SEM **(Ei)** body weight and **(Eii)** testis weight/body weight ratio in the MFN2 WT and MFN2 cKO mice groups (*n* = 6 for each group) at PD 56. N.S. means no significant difference. **: *p* < 0.01.

Exon 6 of the *Mfn2* gene was targeted as the deletion region, in which two LoxP sites were inserted in the mouse genome via CRISPR/Cas9 and homology directed repair (HDR) techniques (Figure 1A). Two guide RNA (gRNA) of the *Mfn2* gene, the donor vector containing the LoxP sites, and Cas9 mRNA were co-injected into fertilized mouse eggs to generate targeted conditional knock-out offspring. F_0_ founder animals were identified by PCR followed by sequence analysis, and these were then bred with WT mice to test the germline transmission and to generate the F_1_ generation. The gRNA sequences used, are as follows with the PAM region underlined:

gRNA1_F (matching the forward strand of the gene): GCA​GGG​ACC​GTG​GTT​TAG​TGG​GG
gRNA1_R (matching the reverse strand of the gene): CAG​GGG​ATC​TAA​TAC​TGT​CCT​GG
gRNA2_F (matching the forward strand of the gene): CGA​CCT​TGG​AGC​AGG​GAC​CGT​GG
gRNA2_R (matching the reverse strand of the gene): GGT​GTA​CAC​AGA​GTA​TAT​CCA​GG.

Mfn2 floxed mice were identified via PCR analysis with Rapid Taq polymerase (Cat. P222-02, 2X Rapid Taq Master Mix, Vazyme, Nanjing, China). A small piece was excised at the tail tip of the mouse and this was lysed with 200 μL digestion buffer (50 mM KCl, 10 mM pH 9.0 Tris-HCl, 0.1% Triton X-100 and 0.4 mg/ml Proteinase K) at 55°C overnight. Then, each sample was incubated at 98°C for 10 min to denature the Proteinase K and centrifuged at 12,000 rpm for 5 min. The supernatant containing the genomic DNA was then used in the PCR assay. Information about the PCR primers used and the expected size of bands obtained, are as follows:

Forward primer: 5′-GAG​GAG​CAT​AAT​AGG​AAA​TGA​GCC-3’
Reverse primer: 5′-GAT​GGA​GAC​TGC​CTA​CTT​CCA​AA-3.
Homozygotes: one band with 205 bp.
Heterozygotes: two bands with 205 bp and 147 bp.
Wild-type (WT) allele: one band with 147 bp.

The PCR mixture consisted of 1 μL mouse tail genomic DNA, 1 μL forward primer (10 μM), 1 μL reverse primer (10 μM), 12.5 μL 2X Rapid Taq Master Mix, and 9.5 μL ddH_2_O. The PCR program involved an initial denaturation step at 95°C for 3 min followed by 32 cycles comprising denaturation at 95°C for 15 s, annealing at 60°C for 15 s, and extension at 72°C for 15 s. This was followed by a final additional extension step at 72°C for 5 min. The PCR products were then separated by gel electrophoresis using a 1.5% agarose gel. This method was used to confirm successful LoxP insertions such that when compared with WT mice, successful insertion resulted in a relatively higher band size due to the increased size of LoxP. Indeed, WT mice showed a single band of 147 bp, heterozygous mice (i.e., Mfn2^loxP/+^) showed two bands at 147 bp and 205 bp, and homozygous mice (Mfn2^loxP/loxP^) demonstrated a single higher band at 205 bp (Figure 1B). The homozygous MFN2 floxed mice were then crossed with Stra8-Cre mice, in which Cre was specifically expressed in male germ cells (Sadate-Ngatchou et al., 2008). Further genetic screening was conducted to select those that also expressed Cre, as shown by the additional band obtained by gel electrophoresis (Figure 1B), whereas the control mice did not express Cre. We could then easily identify the MFN2 cKO mice via the Cre-LoxP system (Gu et al., 1993) and PCR analysis using the following primers:

Forward primer for Stra8-Cre: 5′-GTG​CAA​GCT​GAA​CAA​CAG​GA-3’
Reverse primer for Stra8-Cre: 5′- AGG​GAC​ACA​GCA​TTG​GAG​TC-3’

In all experiments of this study, the control mice were chosen from the same littermate with MFN2 cKO mice. The MFN2 WT control mice were genotyped as Mfn2^fl/fl^ or Mfn2^fl/+^; MFN2 heterozygous mice were genotyped as Mfn2^fl/+^;Stra8-Cre; and the MFN2 homozygous cKO mouse were genotyped as Mfn2^fl/fl^;Stra8-Cre.

### Tissue Collection and Histological Analysis

Male mice were euthanized by Carbon dioxide (CO_2_) inhalation (Niel and Weary 2006) and then the testes were immediately dissected out and fixed in Bouin’s solution (Cat. HT10132, Sigma-Aldrich, United States) for 16 h. The testes were then dehydrated, embedded in paraffin and 7 μm thick sections were prepared and mounted on glass slides. The tissue samples were then de-paraffinized, stained with hematoxylin solution for 90 s and then washed three times with ddH_2_O. They were then mounted with neutral balsam (Cat. G8590, Solarbio, Beijing, China) and imaged via light microscopy. For indirect immunofluorescence (IF) assays, the testes were fixed in 4% paraformaldehyde for 24 h and then they were dehydrated, processed, and sectioned as described above, before they were immunolabeled.

### The Enzyme-Linked Immunosorbent Assay

Blood samples were collected from mice and centrifuged at 10,000 rpm for 10 min. The supernatant (i.e., the serum) was collected for subsequent analysis by ELISA using the mouse testosterone (Cat. AE90626Mu) and luteinizing hormone (Cat. AE90609Mu) kits from AMEKO, Shanghai, China. ELISA assays were performed according to the manufacturer’s instructions.

### Chromosome Spread Assays

Testis tissue was collected from male mice as described above, and the tunica albuginea was removed before the remaining tissue was transferred into a 1.5 ml RNase/DNase Free tube (Cat. AXYMCT150CS, Axygen, Corning, NY, United States) containing 1 ml PBS (Cat. 10010023, Gibco, Thermo Fisher Scientific, MA, United States). Forceps (Cat. HEC7.1, Tweezers Round, Carl Roth, Karlsruhe, Germany) were used to crush the seminiferous tubules and spermatocytes for around 5 min, until the large pieces of tissue had been dissociated. The resulting cell suspension was then filtered into a new 1.5 ml microtube though a 40 μm cell strainer (Cat. 352340, Falcon, Corning, NY, United States). The cells were centrifuged at 3,500 rpm for 3 min, after which the supernatant was discarded, and the pellet was gently resuspended in 1 ml PBS. The cells were washed by centrifugation 1–2 times until the supernatant was clear. After the final wash, the supernatant was removed as usual and the cells were resuspended in 1 ml hypotonic solution (30 mM Tris HCl, 17 mM trisodium citrate, 5 mM EDTA, 50 mM sucrose, pH 8.8) and incubated at room temperature for 30–40 min. The cells were then centrifuged at 3,500 rpm for 3 min and supernatant was discarded, after which the cell pellet was resuspended in 100 μL sucrose (100 mM) and incubated for 5 min at room temperature. The cells were then fixed by adding the same volume of fixative buffer (0.33 M paraformaldehyde, 100 μL 10% Triton X-100, 10 ml PBS and 30 μL 1 N NaOH). Using a PAP pen (Cat. Z377821, Sigma-Aldrich, MO, United States), two circles were drawn on each glass slide to mark the specimen range, after which 20 μL fixed cell suspension was applied to each circle, and the cells were allowed to settle and attach for 3 h at room temperature. Once the liquid inside each circle was completely dry, the slides were stored at −80°C for up to 3 months prior to the immunolabeling experiments.

### Immunofluorescence Assay

Cells or tissue sections that were fixed as described above, were permeabilized in phosphate buffered saline (PBS) containing 0.1% Triton X-100 (Cat. T8787, Sigma-Aldrich, MO, United States; PBST) for ∼15 min. The cells/tissues were then washed twice with PBST, after which they were incubated with blocking buffer consisting of PBS containing 5% BSA (Cat. A1933, Sigma-Aldrich, MO, United States) for 1 h at room temperature. The samples were then incubated with primary antibody (diluted in blocking buffer), overnight at 4°C, after which they were washed for 10 min with three changes of PBS. The samples were then incubated with the appropriate Alexa Fluor-tagged secondary antibody (at 1:500 dilution in blocking buffer) for 1 h at room temperature. At the end of this secondary antibody incubation step, the samples were washed thoroughly with PBST and then washed briefly with distilled H_2_O, after which they were mounted under ProLong Diamond Antifade mountant containing DAPI (Cat. P36962, Invitrogen, MA, United States). After mounting, the samples were incubated at room temperature overnight to cure the mountant, after which they were stored at 4°C prior to being imaged via confocal microscopy. The following primary antibodies were used: rabbit anti-Tom 20 (at 1:200 dilution; Cat. 72610, Cell Signaling Technology, MA, United States), mouse anti-phospho-Histone H2A.X (pSer139) (at 1:300 dilution; Cat. 05–636, Millipore, MA, United States), mouse anti-Sycp3 (at 1:300 dilution; Cat. ab181746, Abcam, Cambridge, United Kingdom), rabbit anti-cleaved-PARP (at 1:200 dilution; Cat. 94885, Cell Signaling Technology, MA, United States), rat anti-H1t (at 1:100 dilution; custom made by Dai·An, Wuhan, China), mouse anti-PLZF (at 1:200 dilution; Cat. sc-28319, Santa Cruz Biotechnology, TX, United States), rabbit anti-PCNA (at 1:200 dilution; Cat. #13110, Cell Signaling Technology, MA, United States), rabbit anti-cleaved-caspase 3 (1:200 dilution; Cat. #9664, Cell Signaling Technology, MA, United States), anti-PEX3 antibody (at 1:200 dilution, Cat. PA5-37012, Thermo Fisher Scientific, MA, United States), anti-TRF1 (at 1:200 dilution; Cat. ab192629, Abcam, Cambridge, United Kingdom), and anti-MLH1 antibody (at 1:200 dilution; Cat. 554073, BD, NJ, United States). The secondary antibodies utilized in these experiments were from Invitrogen, MA, United States and are as follows: Alexa Fluor Plus 488-tagged goat anti-mouse IgG (H + L) highly cross-adsorbed antibody (Cat. A32723), Alexa Fluor Plus 488-tagged goat anti-rabbit IgG (H + L) highly cross-adsorbed antibody (Cat. A32731), Alexa Fluor Plus 594-tagged goat anti-mouse IgG (H + L) highly cross-adsorbed antibody (Cat. A32742), Alexa Fluor Plus 594-tagged goat anti-rabbit IgG (H + L) highly cross-adsorbed antibody (Cat. A32740), and Alexa Fluor Plus 488-tagged goat anti-rat IgG (H + L) highly cross-adsorbed antibody (Cat. A48262). All these secondary antibodies were used at 1:500 dilution. In some experiments, the following fluorescent markers were also used: FITC-conjugated *pisum sativum* agglutinin (FITC-PSA, at 1:500 dilution; Cat. L0770, Sigma-Aldrich, MO, United States), and BODIPY lipid probe (at 1:200 dilution, Cat. D3922, Molecular Probes, Thermo Fisher Scientific, MA, United States).

The mitochondrial membrane potential was evaluated using the JC-1 probe (Cat. T3168, Invitrogen, MA, United States). In brief, spermatocytes were cultured in DMEM/F12 medium with 2 mM JC-1 for 30 min at 37°C, after which they were washed for 3 × 3 min with PBS. The samples were then immediately imaged by confocal microscopy. JC-1 dye exhibits potential-dependent accumulation in mitochondria as indicated by an emission shift in the fluorescence from green (∼529 nm) to red (∼590 nm). Thus, mitochondrial depolarization is indicated by a decrease in the red/green fluorescence intensity ratio. To measure reactive oxygen species (ROS) in spermatocytes, carboxy-H2DCFD (Cat. C400, Invitrogen, MA, United States), which is a fluorescent oxidative stress indicator, was applied in this assay (Takeda et al., 2003). Spermatocytes were pre-treated with DMEM/F12 medium containing 10 mM H_2_O_2_ for 5 min. They were then washed and incubated with DMEM/F12 medium containing 10 µM carboxy-H2DCFD for 30 min at 37°C, after which they were washed 3 × 3 min with PBS and then immediately imaged by confocal microscopy.

### Confocal Imaging

Images of the fluorescently labelled tissue sections or cells were acquired using a ZEISS LSM 900 with Airyscan 2 laser scanning confocal microscope and Hybrid Detectors (HyD). Images were captured with either a Leica HC PL APO CS2 63x/1.4 NA oil immersion objective lens or a Leica HC PL APO ×20/0.7 NA CS2 dry objective lens. Alex Fluor 488, Alex Fluor 546/594, Alex Fluor 647 and DAPI fluorescence was captured with, an argon laser operating at 488 nm, a HeNe laser operating at 561 nm, a HeNe laser operating at 633 nm, or a diode-pumped solid-state laser operating at 405 nm, respectively, using 488 nm excitation/519 nm detection, 552 excitation/575 nm detection, 633 nm excitation/670 nm detection, and 405 nm excitation/461 nm detection, respectively.

### Purification of Male Germ Cells

Male mice germ cells were purified following a well-establish, previously described protocol (La Salle et al., 2009; Gan et al., 2013; Li et al., 2021). In brief, mice were sacrificed and the testes were obtained and decapsulated. The seminiferous tubules were isolated from the other testis tissues by incubation with 0.2% W/V collagenase type I (Cat. 17100017, Gibco, MA, United States) and 1 mg/ml DNase I (Cat. A3778, Applichem, IOWA, United States) in a water bath at 37°C for 10 min, after which the samples were centrifuged at 1,200 rpm for 2 min. The pellet (containing the seminiferous tubules) was then resuspended in 5 ml 0.25% trypsin (Cat.15050057, Gibco, MA, United States) containing 1 mg/ml DNase I at 37°C for 5 min with gentle shaking. The sample was centrifuged at 1,500 rpm for 5 min, after which the pelleted cells were resuspended in 20 ml high glucose DMEM (Cat. 12100046, Gibco, MA, United States) containing 0.5% BSA and filtered through a 40 μm nylon cell strainer (Cat. 352340, Falcon^®^, BD, NJ, United States). The recovered cells in the filtrate were resuspended in 20 ml DMEM containing 0.5% BSA and loaded into a cell separation apparatus (BOMEX Corporate) containing a 2–4% BSA gradient in 600 ml DMEM. After sedimentation for 3 h, the cells were collected into tubes from the bottom of the separation apparatus at a rate of 10 ml/min (i.e., 10 ml/tube). The cell type and purity in each fraction were assessed according to their diameter and morphological characteristics under the light microscope.

### Protein Extraction and Western Blotting Analysis

Purified spermatocytes were collected from WT and MFN2 cKO male C57BL/6 mice testes and suspended in lysis buffer [50 mM HEPES-KOH (pH 7.5), 100 mM KCl, 2 mM EDTA, 10% glycerol, 0.1% NP-40, 10 mM NaF, 0.25 mM Na_3_VO_4_, and 50 mM *ß*-glycerophosphate] supplemented with complete protease inhibitor (Cat. 04693116001, Roche, Basel, Switzerland). The samples were homogenized and centrifuged at 20,000 g for 20 min at 4°C, after which the supernatant was retained for western blotting analysis. The proteins in each sample were separated using 4–12% Bis-Tris gels (Cat. M00652, SurePAGE™, GenScript, Nanjing, China) and a mini protein electrophoresis system (Cat. 1658034, BIO-RAD, CA, United States) following the manufacturer’s instructions. The protein bands were then transferred to polyvinylidene fluoride (PVDF) membranes (Cat. IPVH00010, Immobilon, Millipore, MA, United States) via a Mini Trans-Blot Electrophoretic Transfer Cell (Cat. 1703930, BIO-RAD, CA, United States). The immunoreactive bands were detected and analyzed with a Bio-Rad ChemiDoc MP imaging System (Cat. 12003154, BIO-RAD, CA, United States) in conjunction with the Image Lab Software (Bio-Rad, CA, United States). The relative protein levels in each sample were normalized to *ß*-tubulin or GAPDH to standardize the loading variations. The primary antibodies for immunoblotting were as follows: anti-GAPDH antibody (at 1:2,000 dilution; Cat. #8884, Cell Signaling Technology, MA, United States), anti-β-tubulin antibody (at 1:2,000 dilution; Cat. #2146, Cell Signaling Technology, MA, United States), anti-MFN2 antibody (at 1:1,000 dilution; Cat. #9482, Cell Signaling Technology, MA, United States), anti-PPAR-α antibody (at 1:1,000 dilution; Cat. ab24509, Abcam, Cambridge, United Kingdom), anti-PPAR-β antibody (at 1:1,000 dilution; Cat. SC-74517, Santa Cruz Biotechnology, TX, United States), and anti-PPAR-γ antibody (at 1:1,000 dilution; Cat. SC-7273, Santa Cruz Biotechnology, TX, United States). The secondary antibodies used were: HRP conjugated goat anti-mouse IgG (H + L) cross-adsorbed antibody (at 1:5,000 dilution; Cat. G-21040, Invitrogen, MA, United States) and HRP conjugated goat anti-rabbit IgG (H + L) cross-adsorbed antibody (at 1:5,000 dilution; Cat. G-21234, Invitrogen, MA, United States).

### Quantitative Reverse-Transcription Polymerase Chain Reaction

Total RNA was extracted from isolated pachytene spermatocytes using BSA gradient sedimentation. The extracted RNA was reverse transcribed using the High-capacity cDNA Reverse Transcription kit (Cat. 4368813, Thermo Fisher Scientific, CA, United States) according to the manufacturer’s instructions. Five genes (Sdhb, Uqcrc2, Atp5a1, Ndufv1 and Cox1) and the house-keeping gene Gapdh were quantified using real time-qPCR with SYBR^®^ Green master mix (Cat. 1725124, iTaq™ Universal SYBR^®^ Green Supermix, BIO-RAD, CA, United States), and an Applied Biosystems 7500 Real-time PCR system (Cat. 4351107, Applied Biosystems™, Thermo Fisher Scientific, MA, United States). The following program was used: Activation at 95°C for 3 min (1 cycle) and then 40 cycles of denaturation at 95°C for 15 s and annealing/extension at 60°C for 30 s. The data were normalized to GAPDH and the relative levels of mRNA were quantified using the 2^−ΔΔCt^ method. The primers used in this assay are as follows:

*Mfn2* Forward primer: 5′- GCT​CCT​GAA​GGA​TGA​CCT​CG-3’
*Mfn2* Reverse primer: 5′-CGT​CTG​CAT​CAG​CGT​GGA​CTC-3’
*Sdhb* Forward primer: 5′-AAT​TTG​CCA​TTT​ACC​GAT​GGG​A-3’
*Sdhb* Reverse primer: 5′-AGC​ATC​CAA​CAC​CAT​AGG​TCC-3’
*Uqcrc2* Forward primer: 5′-AAA​GTT​GCC​CCG​AAG​GTT​AAA-3’
*Uqcrc2* Reverse primer: 5′-AAA​GTT​GCC​CCG​AAG​GTT​AAA-3’
*Atp5a1* Forward primer: 5′-TCT​CCA​TGC​CTC​TAA​CAC​TCG-3’
*Atp5a1* Reverse primer: 5′-CCA​GGT​CAA​CAG​ACG​TGT​CAG-3’
*Ndufv1* Forward primer: 5′-TTT​CTC​GGC​GGG​TTG​GTT​C-3’
*Ndufv1* Reverse primer: 5′-GGT​TGG​TAA​AGA​TCC​GGT​CTT​C-3’
*Cox1* Forward primer: 5′-GTG​CTG​GGG​CAG​TGC​TGG​AG-3’
*Cox1* Reverse primer: 5′-TGG​GGC​CTG​AGT​AGC​CCG​TG-3’
*Gapdh* Forward primer: 5′-GGC​AAA​TTC​AAC​GGC​ACA​GT-3’
*Gapdh* Reverse primer: 5′-GGC​CTC​ACC​CCA​TTT​GAT​GT-3’

### RNA Sequencing and Data Analysis

RNA sequencing was performed with an Illumina HiSeq PE150 by Novogene Corp. Inc. (Beijing, China). Total RNA was purified from isolated pachytene spermatocytes using Trizol reagent (Cat. 15596026, TRIzol™, Thermo Fisher Scientific, CA, United States) according to the manufacturer’s instructions. The RNA quality and quantity were evaluated via Bioanalyzer (Agilent Co.) and Qubit (Life Technologies), respectively, and normalization was performed using Novomagic software (Novogene Corp. Inc.) to generate the number of reads in fragments per kilobase million (FPKM). Genes having an average FPKM >1 in at least one condition were used in subsequent scatterplot analyses. Differential expression analysis was performed using DESeq2, and genes with an adjusted *p*-value < 0.05 were considered as being differentially expressed. Expression pattern clusters were generated by the unsupervised hierarchical clustering analysis and K-means clustering algorithm using R. DAVID, and IPA were used to reveal the Gene Ontology (GO) and Kyoto Encyclopedia of Genes and Genomes (KEGG) analysis.

### Quantification of Mitochondrial DNA Copy Number in Spermatocytes

To quantify the mtDNA copy number in spermatocytes, total DNA was extracted from mouse spermatocytes using the GeneJET genomic DNA purification Kit (Cat. K0721, Thermo Fisher Scientific, CA, United States) according to the manufacturer’s instructions. The total DNA was then quantified using a NanoDrop 2000 spectrophotometer (Cat. ND-2000, Thermo Fisher Scientific, CA, United States). The Mt-Rnr2 (16S ribosomal RNA) was used to determine the mtDNA copy number and *β-actin* was selected as the house-keeping gene. PCR reactions were performed with the 7500 Real-Time PCR System (Applied Biosystems) using an initial activation step at 95°C for 3 min, followed by 40 cycles of denaturation at 95°C for 15 s and annealing/extension at 60°C for 30 s. The data were normalized to *ß*-*actin* and the relative mRNA levels were quantified using the 2^−ΔΔCt^ method. The primers used were as follows:

*Rnr2*_F: 5′-TGC​CTG​CCC​AGT​GAC​TAA​AG-3’
*Rnr2*_R: 5′-GAC​CCT​CGT​TTA​GCC​GTT​CA-3’
β-*actin* _F: 5′- GGC​TGT​ATT​CCC​CTC​CAT​CG-3’
β-*actin* _R: 5′-CCA​GTT​GGT​AAC​AAT​GCC​ATG​T-3’

### Statistical Analysis

The data were analyzed with Minitab version 18 (Minitab Inc. State College, PA, United States) using the Student’s *t*-test or one-way ANOVA followed by Tukey’s test. *p*-values less than 0.05 were considered to be statistically significant. Graphs were generated using Microsoft Excel and figures were prepared with CorelDraw version X8 (Corel Corp., Ottawa, ON, Canada).

## Results

### MFN2 Is Essential for Male Spermatogenesis

The *Mfn2*-floxed and Stra8-Cre cKO mice were genotyped (Figure 1B). The higher band at 205 base-pair (bp) represents a successful loxP insertion band in Mfn2 exon 6 region, while the lower band at 147 bp represents a wild-type Mfn2 band in exon 6 region (Figure 1B). Here, we utilized *Mfn2^fl/fl^* or *Mfn2^fl/+^* animals as MFN2 wild-type controls and they were labeled as MFN2 WT in the following text and figures. In general, we sacrificed animals from the same littermate and made compare among these animals in all experiments. At first, the levels of *Mfn2* exon6 mRNA and MFN2 protein were determined in spermatocytes from MFN2 WT and MFN2 cKO mice via qPCR (Figure 1Ci) and western blot analysis (Figure 1Cii). For the mRNA level of *Mfn2* exon 6, it showed an extremely low expression in MFN2 cKO group, which indicates that there is no aberrant mRNA in the cytoplasm of MFN2 deficient spermatocytes (Figure 1Ci). For MFN2 protein expression, as shown in the gel, the MFN2 deficient spermatocytes had low levels of MFN2 protein when compared with the MFN2 WT spermatocytes, which suggests that much of the MFN2 protein had successfully been knocked out in the spermatocytes of the MFN2 cKO line. The testis of each of two representative MFN2 WT and MFN2 cKO mice were excised at postnatal day (PD) 56 (Figure 1D) and the body weight, testis/body weight ratio were determined (Figure 1E). The data show that the growth and body weight between MFN2 WT controls and MFN2 cKO mice are showed no significant differences (Figure 1Ei), while the testis in the MFN2 cKO mice were clearly smaller than the testis of the MFN2 WT controls (Figure 1D). This is also reflected in the significantly lower testis/body weight ratio in the MFN2 cKO group when compared with the controls (Figure 1E). The testis size of MFN2 heterozygous animals were also analyzed, and there is no difference in testis size between MFN2 WT controls and MFN2 heterozygous mice (Supplementary Figure S1A).

**FIGURE 2:**
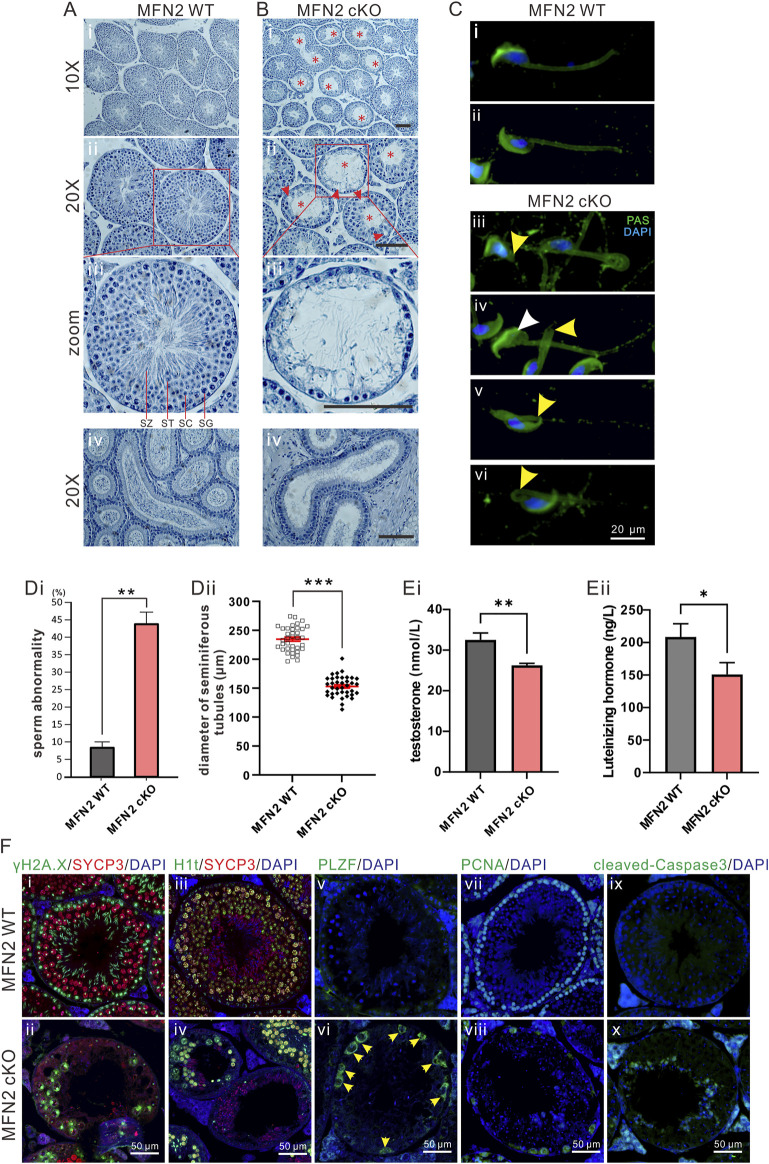
The MFN2 cKO mouse exhibits abnormal spermatocyte differentiation. **(A,B)** Testes sections acquired at postnatal day (PD) 56 from **(Ai–Aiii)** MFN2 WT and **(Bi–Biii)** MFN2 cKO mice, were stained with hematoxylin. In the MFN2 cKO testes, the red asterisks indicate abnormal tubules in which the majority of the spermatogonia (SG), spermatocytes (SC) and post-meiotic spermatids (ST) were absent. SZ: spermatozoon. **(Aiv,Biv)** Epididymis histology sections were also stained with hematoxylin reagent. Scale bars are 100 μm. **(C)** Spermatozoa were extracted from the epididymis of **(Ci,Cii)** MFN2 WT and **(Ciii–Cvi)** MFN2 cKO mice at PD 35. They were then spread and fixed on glass slides, before being labeled with FITC-Periodic Acid-Schiff (PAS; in green) and DAPI (in blue). In the MFN2 cKO examples, the yellow arrowheads in **(Ciii–Cvi)** indicate abnormal spermatozoa containing a truncated or bent tail, and the white arrowhead in **(Civ)** indicates a spermatozoon with an abnormal nucleus. Scale bar is 20 μm. **(Di)** Bar graph to show the ratio of sperm abnormality in the MFN2 WT and MFN2 cKO groups, *n* = 223 and *n* = 300 spermatozoa were quantified in MFN2 WT and MFN2 cKO groups, respectively. **(Dii)** Individual dot plot to show the diameter of seminiferous tubules in MFN2 WT and MFN2 cKO testes. **(E)** Bar graphs to show the levels of **(Ei)** testosterone and **(Eii)** luteinizing hormone (LH) in the serum of MFN2 WT and MFN2 cKO mice, which were measured via ELISA from *n* = 3 mice in each group. In **(Di–Eii)**, the data represent the mean ± SEM of three independent experiments and *p < 0.05, **p < 0.01. **(F)** Testis tissue sections from MFN2 WT and cKO groups were immunolabelled with various primary antibodies (γH2A.X labels double-strand breaks, SYCP3 labels meiotic spermatocytes, H1t labels mid-pachytene spermatocytes, PLZF labels undifferentiated cells, PCNA labels proliferative cells, cleaved-Caspase3 labels apoptotic cells and DAPI labels nuclei) and they were co-stained with DAPI (blue). Images were acquired via fluorescent microscopy. In **(Fvi)**, the yellow arrowheads indicate PLZF-positive cells. Scale bars are 50 μm.

Histological analysis of the testis tissue from WT and MFN2 cKO mice at PD 56 demonstrated the severe negative effect on the inner structure found in the latter (Figures 2A,B). A comparison of the high magnification views (Figures 2Aiii, 2Biii) shows that in the WT testes, differentiated cells could clearly be observed, but in the MFN2 cKO testes, there were obvious abnormalities. Indeed, in the former, from the outside to the central area of the seminiferous tubules, well-defined spermatogonia (SG), spermatocytes (SC), spermatids (ST) and spermatozoon (SZ) could be clearly distinguished (Figures 2Aiii). However, in MFN2 deficient mice the majority (∼70%; Figures 2Bi) of the seminiferous tubules were devoid of any cells (Figures 2Biii). The histological studies in MFN2 heterozygous mice showed no obvious effect in seminiferous tubules and spermatocytes (Supplementary Figures S1B). Moreover, the meiotic spermatocytes in different meiotic stages, including SC, ST and SZ, could be clearly detected from the histological images (Supplementary Figures S1B). Furthermore, we showed that epididymis tissue from WT mice contained many mature spermatozoa (Figure 2Aiv), whereas only a limited number could be detected in the epididymis of the MFN2 deficient mice (Figure 2Biv). FITC-PAS (green) was applied to visualize the acrosome of the spermatozoa in the WT and cKO groups, however other parts of the sperm were also labeled due to their highly sticky nature (Figure 2C). The nuclei were labeled with DAPI (blue). The percentage of abnormal spermatozoa were then quantified (Figure 1Di). The data showed that while WT mice contained 8.3% abnormal mature sperm in the epididymis, the MFN2 cKO mice exhibited 43.8% abnormal sperm and these had a variety of phenotypes including a folded or broken tail, and an abnormal nucleus (Figures 2C, 2Di). The diameter of the seminiferous tubules of MFN2 cKO mice is reduced significantly (∼151 μm) when it compared with the seminiferous tubules from controls mice (∼235 μm; Figure 2Dii). As it is widely known that the sex hormones are crucial for testis development, so the levels of testosterone and luteinizing hormone were also determined in the MFN2 WT and MFN2 cKO mice via ELISA (Figures 2Ei,2Eii). Our data showed that both hormones were significantly reduced in the MFN2 cKO mice when compared with the MFN2 WT controls.

MFN2 WT and cKO testes were immunolabeled with various antibodies to determine the effect of MFN2 on spermatocyte differentiation. The testes were dual-immunolabeled with antibodies to γH2A.X and SYCP3. The former is a marker for DNA double strand break (DSB) repair, which aggregates in the sex chromosomes during meiosis in the pachytene stage, whereas the latter labels spermatocytes which are undergoing meiosis (Li et al., 2019). We showed that in WT testis, the majority of SYCP3-positive spermatocytes show aggregated γH2A.X signals in the sex bodies (Figure 2Fi), which suggests they have already passed into the pachytene or diplotene stages. However, in MFN2 cKO spermatocytes, γH2A.X was retained largely on the autosomes, and almost none of the spermatocytes had aggregated γH2A.X signals in the sex body (Figure 2Fii). This might indicate that the meiotic process from the zygotene to pachytene stages is abnormal in the MFN2 cKO spermatocytes. MFN2 WT and cKO testes were also immunolabeled with an H1t antibody, which is a specific marker that only starts to be expressed after the mid-pachytene stage (Li et al., 2021). It gave a very nice labeling pattern for WT pachytene or diplotene spermatocytes (Figure 2Fiii), but its signal was considerably lower in the MFN2 deficient spermatocytes (Figure 2Fiv). Indeed, some of the seminiferous tubules in the MFN2 cKO mice had almost no H1t positive spermatocytes (Figure 2Fiv). These data again indicate that in the MFN2 cKO mice, the spermatocytes might not progress to the mid-pachytene stage due to abnormal meiosis. Promyelocytic leukemia zinc-finger (PLZF) is a marker of undifferentiated cells. We showed that at PD 56, no PLZF-positive spermatogonia were detected in WT mouse testis (Figure 2Fv), whereas there was obvious PLZF-positive spermatogonium in the cKO group (Figure 2Fvi). These data indicate that spermatogenesis was retarded in MFN deficient testis tissue. We also examined if cells were undergoing proliferation or apoptosis in the WT and MFN2 cKO testis by immunolabeling with the PCNA (Figures 2Fvii,2Fviii) and cleaved caspase 3 (Figures 2Fix,2Fx) antibodies, respectively. In the WT controls, the PCNA antibody labeled every spermatogonia in the base area of the seminiferous tubules, whereas in the MFN2 cKO animals only a few spermatogonium were PCNA positive (Figures 2Fvii,2Fviii). In contrast, cleaved caspase 3 labeling showed that no cells were undergoing apoptosis in the WT controls, whereas apoptotic signals were apparent in the MFN2 cKO testis (Figures 2Fix,2Fx). These data indicate that in the MFN2 cKO testis, the spermatogonium are in defective.

### MFN2 Is Required for Meiosis in Spermatocytes

**FIGURE 3:**
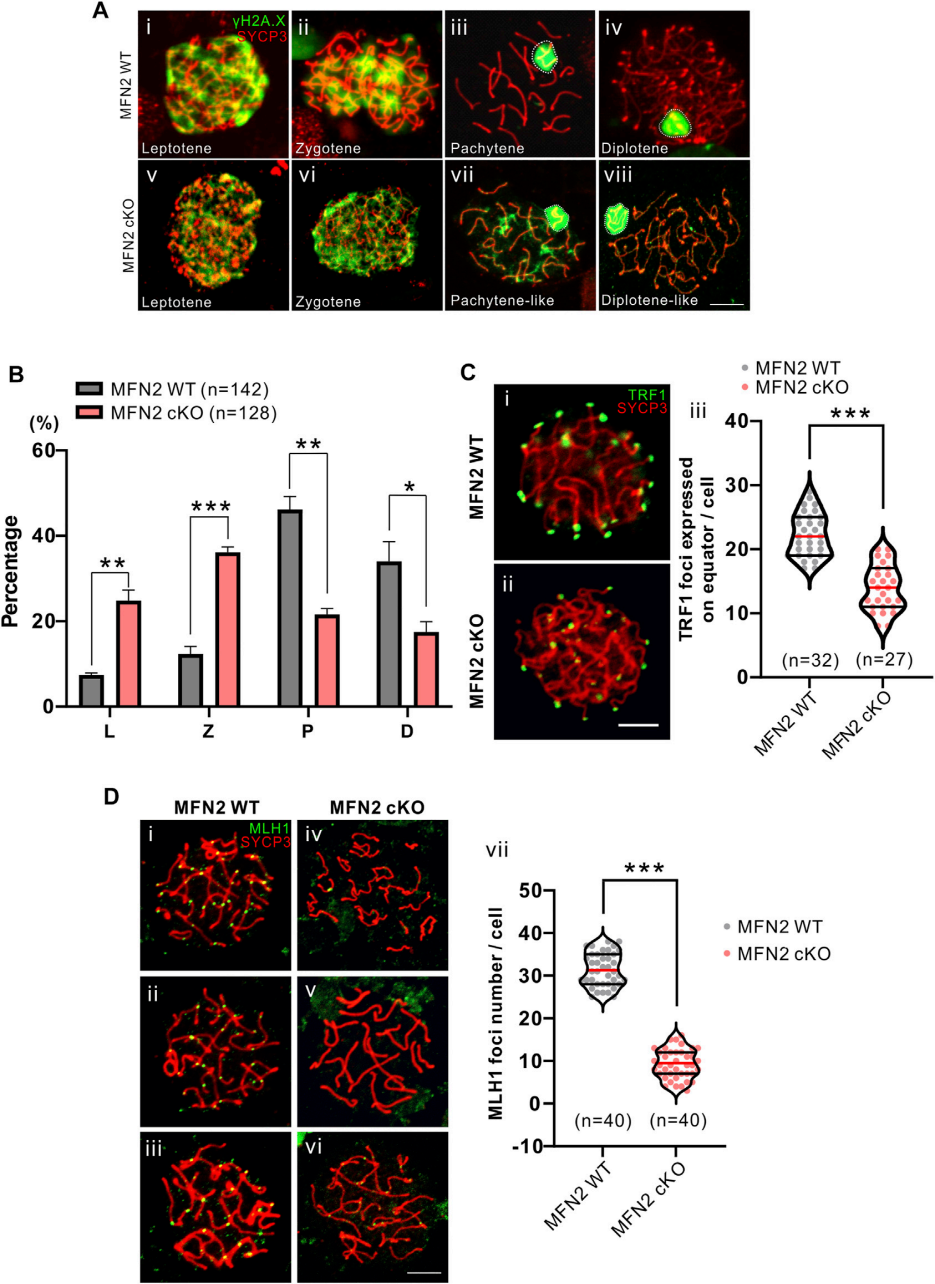
Comparison of meiotic chromosome spreads from MFN2 WT and MFN2 cKO spermatocytes. **(A, D)** Harsh and **(C)** mild chromosome spreads were prepared before immunolabeling procedures were conducted with the γH2A.X, ΤRF1, or MLH1 antibodies (in green) and the Sycp3 antibody (in red). The latter was used to label meiotic chromosomes. **(A)** Spermatocytes are shown at different stages. **(B)** The percentage of spermatocytes at these stages (i.e., L: leptotene, Z: zygotene, P: pachytene, and D: diplotene) were quantified in the MFN2 WT and MFN2 cKO groups. **(C)** The telomeres were immunolabelled with an anti-TRF1 antibody. **(Ci,Cii)** Images were captured by confocal microscopy. **(Ciii)** Three optical sections in the middle of the z-stack were used to quantify the TRF1 foci, which were expressed on the equator. 32 MFN2 WT spermatocytes and 27 MFN2 cKO spermatocytes were quantified in this experiment. **(D)** Pachytene stage spermatocytes in the **(Di-Diii)** MFN2 WT and **(Div-Dvi)** MFN2 cKO groups were immunolabelled with the anti-MLH1 (in green) and anti-SYCP3 (in red) antibodies. **(Dvii)** The number of MLH1 foci were quantified in the MFN2 WT and MFN2 cKO spermatocytes (*n* = 40 for each group). Scale bars are 5 μm *: *p* < 0.05; **: *p* < 0.01; ***: *p* < 0.001.

We compared the expression of γH2A.X in meiotic chromosome spreads prepared from WT and cKO spermatocytes (Figure 3) and found that it is expressed in autosomes and sex chromosomes in both groups at the leptotene and zygotene stages (Figures 3Ai,3Aii, 3Av,3Avi). In the WT group at the pachytene stage, γH2A.X was expressed in the sex chromosomes but not in the autosomes (Figure 3Aiii), whereas at the equivalent stage (called pachytene-like) in the MFN2 cKO group, γH2A.X was still expressed in the autosomes as well as in the sex body (Figure 3Avii). Indeed, in the cKO group, the γH2A.X signal could still be detected in autosomes even at the diplotene-like stage (Figure 3Aviii). We also quantified the number of spermatocytes at the different stages by distinguishing the SYCP3 and γH2A.X expression patterns (Figure 3B). MFN2 cKO group showed a higher percentage of spermatocytes in leptotene and zygotene stages while present a significant lower percentage for both pachytene and diplotene stages when it compared with WT group (Figure 3B). Moreover, the telomere dynamics and the touching ability with nuclear membrane were also analyzed by immunolabeling the telomeres (in green, Figure 3C). In the WT group, TRF1 foci were clearly expressed around the equator, indicating good attachment between the telomeres and nuclear membrane (Figure 3Ci). In contrast, in the MFN2 cKO group, some of the telomere foci were in the central region of spermatocytes, indicating that they were not well attached to the nuclear membrane (Figure 3Cii). Quantification of the numbers of foci showed that the average number of TRF1 foci at the equator was 21.8 in the WT group and just 15.5 in the MFN2 cKO group (Figure 3Ciii). These data suggest that MFN2 is involved in the regulation of telomere-driven chromosome dynamics during meiosis. We then analyzed the expression of MLH1 in the WT and cKO groups to test the chromosome cross-over process from the late-pachytene to early-diplotene stages (Figure 3D). In WT spermatocytes, MLH1 foci were clearly observed on the chromosomes (Figures 3Di-Diii), whereas in the cKO group, only a few MLH1 foci were visualized (Figures 3Div-Dvi). This suggests that the cross-over process was blocked in MFN2 cKO spermatocytes.

### MFN2 Deficiency Leads to the Aggregation of Mitochondria and Disrupted Respiratory Chain

**FIGURE 4:**
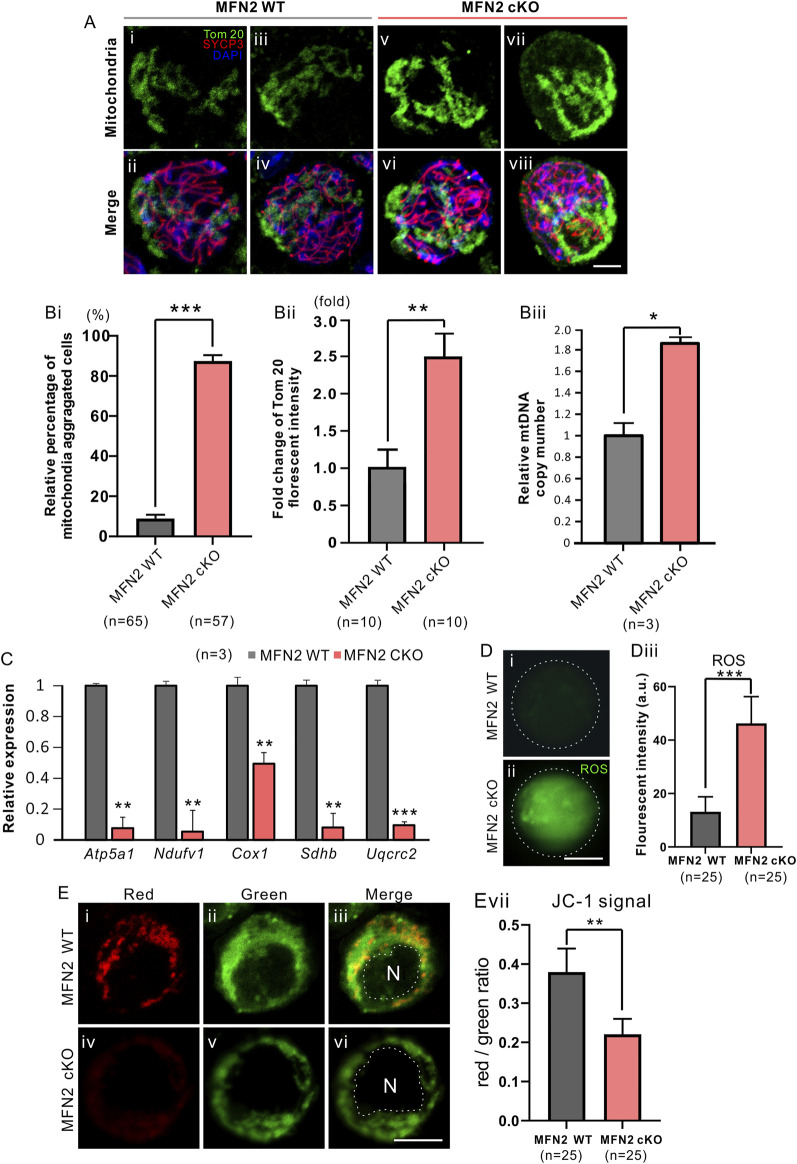
MFN2 deficiency leads to the aggregation of mitochondria in spermatocytes. **(A)** Testis tissue from (Ai-Aiv) MFN2 wild-type (WT) and (Av-Avi) MFN2 cKO animals were dissociated at PD 35, and the spermatocytes were collected and cytospin onto glass slides. They were then fixed and permeabilized before being immunolabeled with anti-Tom20 and -SYCP3 antibodies, to visualize the mitochondria and spermatocyte chromosomes, respectively. **(B)** Quantification of **(Bi)** the relative percentage of mitochondria-aggregated spermatocytes **(Bii)** fold change in Tom 20 fluorescent intensity and **(Biii)** relative mitochondria DNA (mtDNA) copy number, in the MFN2 WT and MFN2 cKO groups. The *n* = 3 is under each graph. **(C)** Bar chart to show the expression of five respiratory chain genes, which were measured by RT-qPCR. **(D)** The level of ROS was visualized in the **(Di)** MFN WT and **(Dii)** MFN2 cKO spermatocytes by labeling with carboxy-H2DCFD; the images were acquired by confocal microscopy. **(Diii)** Bar chart to show the quantification of ROS in *n* = 25 spermatocytes in each of the MFN WT and MFN2 cKO groups, determined from the fluorescent intensity. **(E)** The mitochondrial membrane potential was measured via JC-1 staining of the in the **(Ei-Eiii)** MFN WT and **(Eiv-Evi)** MFN2 cKO groups, N means nucleus. **(Evii)** Bar chart to show the red/green fluorescent intensity ratio in each group (*n* = 25 for each). For all the graphs **(Bi-Biii, C, Diii, Evii)**, the data represent the mean ± SEM and the Students’ *t*-test was applied to analyze the data between each two groups, such that *: *p* < 0.05; **: *p* < 0.01; and ***: *p* < 0.001. Scale bars are 10 μm.

We evaluated the distribution of mitochondria in MFN2 WT and MFN2 cKO spermatocytes by immunolabeling them with Tom 20, a marker for the mitochondrial outer membrane (Figure 4). We showed that the WT spermatocytes demonstrated a homogenous, relatively well-spread distribution of mitochondria (Figures 4Ai-4Aiv), whereas in the MFN2 cKO group, the mitochondria had a more aggregated localization (Figures 4Av-4Aviii). Indeed, more than 80% of the MFN2 cKO spermatocytes showed this mitochondrial aggregation phenotype, whereas only around 8% of the WT spermatocytes showed a similar effect (Figure 4Bi). In addition, the expression of Tom 20 was approximately 2.5-fold higher in MFN2 deficient spermatocytes than in WT spermatocytes (Figure 4Bii), and the mtDNA copy number was also significantly higher in the MFN2 cKO group than in the WT controls (Figure 4Biii). We also assessed the mitochondria respiratory chain by evaluating the level of expression of *Atp5a1*, *Ndufv1*, *Cox1*, *Sdhb* and *Uqcrc2*, which are representative of complex I-V, respectively. We found that when compared with the MFN2 WT controls, the MFN2 cKO spermatocytes exhibited a significantly lower level of expression of all these genes (Figure 4C). This indicates that the respiratory function of the mitochondria in the MFN2 cKO spermatocytes was significantly disrupted. In addition, spermatocytes in the MFN2 cKO group had a significantly higher level of ROS when it compared with the WT controls (Figure 4D). Moreover, the red/green ratio of JC-1 staining was significantly decreased in MFN2 cKO spermatocytes when compared with the WT controls (Figure 4E). This suggests that the mitochondrial membrane potential was depolarized after deletion of MFN2. Together, these data suggest that in MFN2 cKO spermatocytes, the function of the mitochondria is disrupted.

### MFN2 Is Involved in the Regulation of the Peroxisome Proliferator-Activated Receptor Signaling Pathway

**FIGURE 5:**
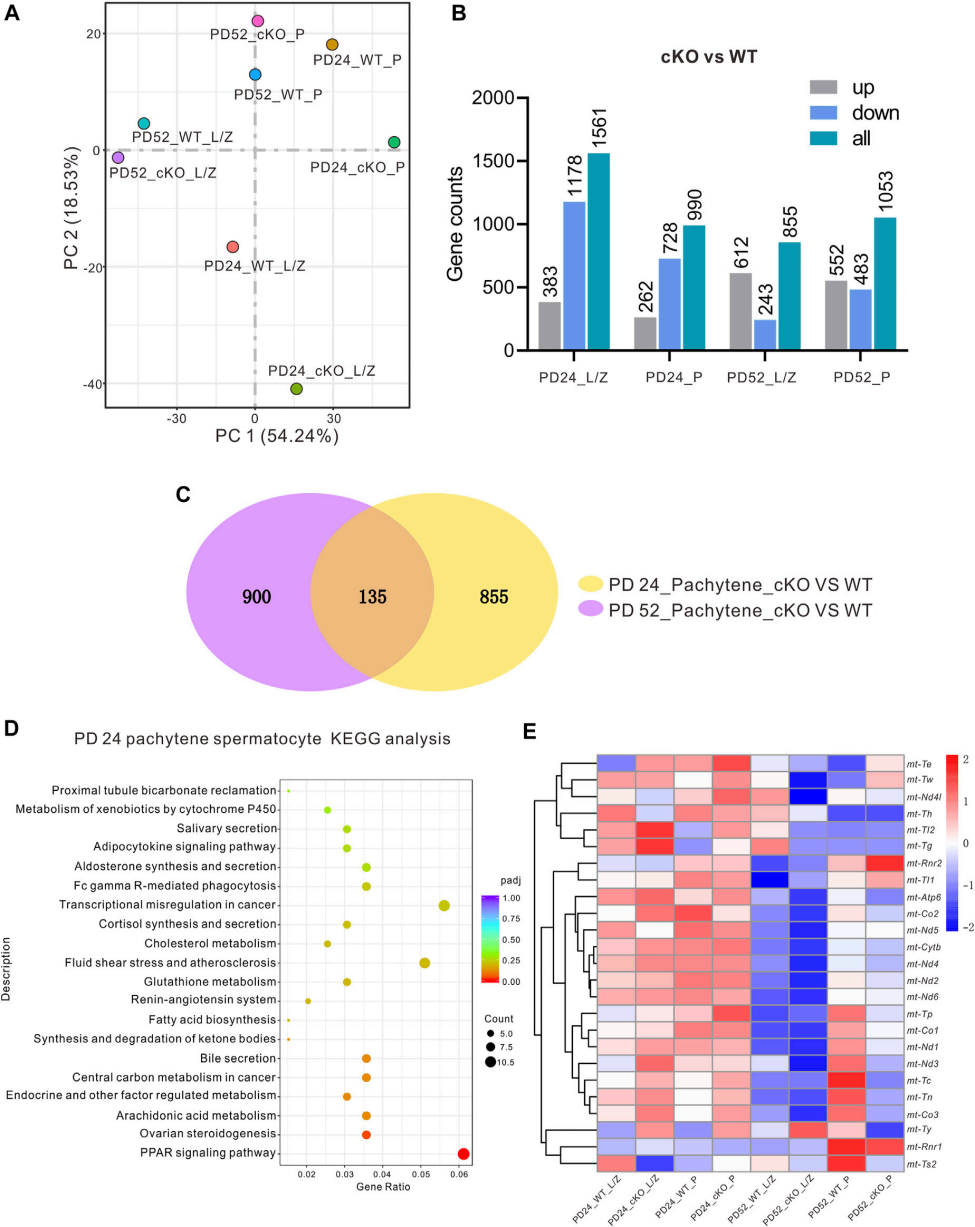
RNA-seq and KEGG analysis for the MFN2-deficient spermatocytes. Leptotene/zygotene (L/Z) and pachytene (P) spermatocytes from PD 24 and PD 56 MFN2 WT and MFN2 cKO mice were collected by BSA density gradient isolation, and the RNA was extracted and RNA-seq analysis was conducted. **(A)** The data distribution of the eight samples after running RNA-seq. **(B)** The number of up-/down-regulated genes was quantified in the different samples groups in the MFN2 cKO group compared to the MFN2 WT. **(C)** Venn diagram to illustrate the numbers of genes changed in the PD 24 and PD 56 pachytene spermatocytes after knocking out MFN2. **(D)** KEGG analysis of the MFN2 deficient PD 24 pachytene spermatocytes. **(E)** Heat map to show the expression of the mitochondria related genes in the eight sample groups.

RNA-seq analysis was applied to isolated pachytene (P) spermatocytes and leptotene or zygotene (L/Z) spermatocytes isolated from the MFN2 WT controls and MFN2 cKO mice at PD 24 and PD 52 (Figure 5). In total, eight groups of data were collected. First, it was obvious that when comparing the WT and MFN2 cKO groups more differences were found in the genes at PD 24 than at PD 52 (Figure 5A). Indeed, 262 genes were up-regulated and 728 genes were down-regulated after MFN2 deletion in the PD 24 pachytene spermatocytes, whereas 552 genes were up-regulated, and 483 genes were down-regulated after MFN2 deletion at PD 52 (Figure 5B). As shown in the Venn diagram (Figure 5C), we screened out 135 genes, which had the same change trend in the PD 24 and PD 56 pachytene spermatocytes after MFN2 deletion. We also showed by KEGG analysis (Figure 5D) and GO analysis (data not shown) that the PPAR signaling pathway was substantially changed in MFN2 cKO spermatocytes. The heatmap (Figure 5E) summarizes the changes observed in 25 mitochondrial genes among the eight groups. We found that several mitochondrial genes, including mt-Tl2, mt-Tg, mt-Atp6, mt-Co2, mt-Cytb, and mt-Nd4, were upregulated in MFN2 cKO animals at PD 24 but downregulated at PD 52 (Figure 5E).

**FIGURE 6:**
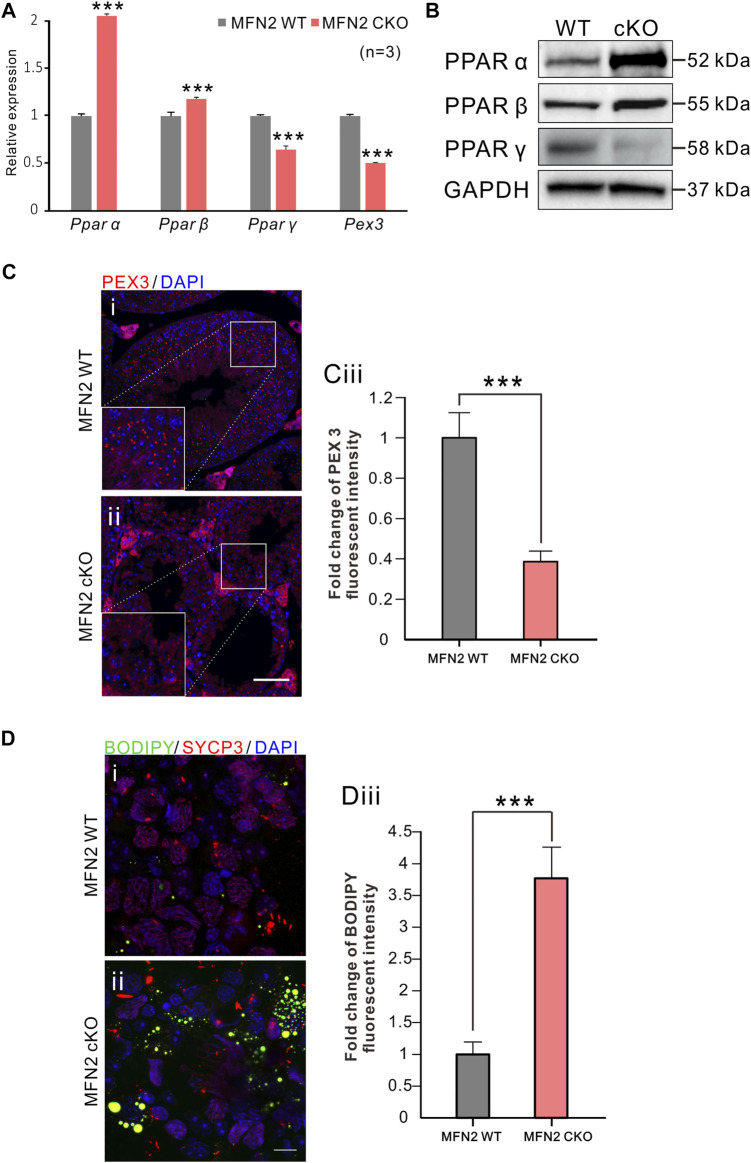
PPAR signaling were significantly altered in MFN2 deficient spermatocytes. **(A)** mRNA expression levels of *Pparα*, *Pparβ*, *Pparγ* and Pex3 were measured in MFN2 WT and MFN2 cKO spermatocytes via RT-qPCR. The data represent mean ± SEM and the cKO values were normalized to the WT controls. This data was quantified from *n* = 3 independent experiments. ***: *p* < 0.001.**(B)** The PPAR family protein expression levels were analyzed via western blot analysis using isolated spermatocytes. GAPDH was used as the loading control. **(Ci-Cii)** Testes sections were labeled with the anti-PEX3 antibody (in red) and co-labeled with DAPI (in blue) to label peroxisomes and cell nuclei, respectively. Scale bar is 50 μm. **(Ciii)** Bar chart to show the quantification of PEX 3 signal fluorescent intensity in MFN2 WT and cKO testes. **(Di-Dii)** Testis cells were immunolabeled with the anti-SYCP3 antibody to label spermatocytes (in red) and then co-labeled with BODIPY (in green) and DAPI (in blue) to label neutral lipid droplets and the cell nuclei, respectively. Scale bar is 10 μm. **(Diii)** Bar chart to show the quantification of BODIPY fluorescent intensity in MFN2 WT and cKO testicle cells. **(Ciii and Diii)** these data represent the mean ± SEM and the Students’ *t*-test was applied to analyze the data between each two groups, such that ***: *p* < 0.001.

We also investigated the expression of various *Ppar* genes by RT-qPCR, and found that *Ppar α* and *Ppar β* were both significantly up-regulated in MFN2 cKO spermatocytes, whereas *Ppar γ* and *Pex3* were significantly down-regulated in this group (Figure 6A). *Pex3* is involved in peroxisome biosynthesis and integrity, the product of this gene is able to assemble membrane vesicles before the matrix proteins are translocated (Jansen and van der Klei 2019). Western blot analysis of the PPAR proteins indicated that similar to the genes, PPAR α and PPAR *ß* were more strongly expressed in MFN2 cKO spermatocytes than in the MFN2 WT group (Figure 6B). Interestingly, the PPAR γ showed almost no expression after MFN2 deletion (Figure 6B). Likewise, we showed that the level of peroxisome expression was decreased significantly in the testis of MFN2 cKO mice, with only ∼0.39-fold of PEX3 fluorescent intensity than that in the controls (Figure 6C). Finally, we assessed the amount of lipid in the WT and MFN2 cKO groups. BODIPY was used to stain the neutral lipid droplets in isolated testicle cells and SYCP3 was used to label the spermatocytes (Figure 6D). Our data showed that there were significant higher numbers of mobile lipid droplets in the MFN2 cKO group, the fluorescent intensity for BODIPY signals showed around ∼3.77-fold change in MFN2 cKO group than that in the MFN2 WT controls. (Figure 6D).

## Discussion

Although mitochondrial dynamics have been investigated for several decades, this topic has gained in popularity in recent years. In the present study, we generated an MFN2 conditional knock-out line via the *Stra8*-driven expression of Cre, which specifically knocked out the *Mfn2* gene in the germ cells of male mice. The *Stra8*-Cre transgenic mouse line was utilized in this project as the Cre expression only in males beginning at postnatal day 3 in early-stage spermatogonia. The Cre is also reported to be detected through preleptotene-stage spermatocytes (Sadate-Ngatchou et al., 2008). We found that in MFN2-deficient spermatocytes, the mitochondria were aggregated in the cytosol and the meiotic process was disrupted such that the cells could not move from the zygotene stage to the pachytene stage. We also isolated spermatocytes at high purity for RNA-seq analysis and found that PPAR signaling was significantly affected after genetic deletion of the *Mfn2* gene. Furthermore, we found that lipid metabolism was abnormal in MFN2 deficient cells.

The first mitofusin knock-out mice were reported in 2003 by David C. Chan’s group. They generated both Mfn1 and Mfn2 global knock-out mice and found that the heterozygous animals were fully viable and fertile, but the homozygous mutants showed embryonic lethality (Chen et al., 2003). They utilized western blotting and demonstrated that either MFN1 or MFN2 were fully knocked-out in the homozygous mutants. In our study, the cKO line we prepared was also highly efficient as most of the MFN2 protein was silenced in purified cKO spermatocytes. We used this approach to evaluate the efficiency of our mutant line as the KO was conditional for spermatocytes only rather than in the whole testis tissue. We found that for our western blotting analysis, instead of losing the MFN2 band completely, the cKO group still showed a very weak band for MFN2 expression. This might be because the Cre-LoxP system is not 100% efficient, and so a few spermatocytes might still express MFN2. In addition, the gravity sedimentation system used to purify the spermatocytes has some limitations in that it is a challenge to obtain a 100% pure sample without contamination from some somatic cells. Unlike the spermatocytes, these cells were not genetically edited and so they might have led to the low expression band of MFN2 on the western blot.

We found that MFN2 deficient animals had empty seminiferous tubules. In addition, the apoptotic marker (cleaved-Caspase 3) was elevated, which indicates that apoptosis was higher in the MFN2 deficient spermatocytes compared with the controls. The previous studies demonstrated the crosstalk between somatic cells and germ cells during spermatogenesis (Wu et al., 2020), spermatogenic cycle has an effect on stem Leydig cell proliferation and differentiation (Guan et al., 2019). Another study reported spermatogenesis involves cell–cell interactions and gene expression orchestrated by luteinizing hormone which controls the production of testosterone by Leydig cells (Oduwole et al., 2021). Our data showed decreased luteinizing hormone and testosterone levels in MFN2 cKO mice, which might indicate disrupted functions of somatic cells, such as Sertoli cell and Leydig cell, in MFN2 cKO testis.

We also found, from dynamic changes in the DNA double-strand break repair marker γH2A.X and the mid-pachytene stage specific marker H1t, that the majority of MFN2 deficient spermatocytes failed to enter the pachytene stage. This indicates that MFN2 deficient spermatocytes were likely to have been undergoing apoptosis due to abnormal meiosis, which might help to explain the abnormal testicle morphology observed. We also showed that the number of spermatocytes in the middle of the seminiferous tubules was negligible. These results are consistent with previous studies on MFN family proteins, which demonstrated that a deficiency in MFN leads to spermatocyte apoptosis (Varuzhanyan et al., 2019). Our data also showed that MFN2 cKO animals were positive for PLZF, which is an undifferentiation marker in spermatogonia, whereas WT animals were negative for PLZF. This might indicate that the spermatogonia were affected after MFN2 deletion as normally these cells do not express PLZF at PD 56. The spermatogonia were also evaluated with the proliferation marker PCNA. The WT spermatogonia were actively undergoing mitosis and thus they were positive for the proliferation marker. However, spermatogonia from the MFN2 cKO mice demonstrated poor PCNA expression, which helps to confirm that the normal cell cycle and differentiation activity of these cells was negatively affected in the cKO group. These data are consistent with those from a previous study in which MFN2 was knocked-out via Ddx4-driven Cre recombination (Chen et al., 2020).

We also assessed the meiotic process in the spermatocytes from MFN2 cKO mice. Using meiotic chromosome spreads assay, we found that the γH2A.X signal was retained in the autosomes during the pachytene-like and diplotene-like stages in the MFN2 cKO group. These results are consistent with the testes tissue section immunolabeling data shown in Figure 2. SYCP3-positive cells are germ cells, which are undergoing meiosis, and although most of the spermatocytes exhibited concentrated γH2A.X signals in the sex chromosome in the WT group, this phenomenon was not seen in the MFN2 deficient spermatocytes as γH2A.X were expressed in both the autosomes and sex chromosomes. A previous study also concludes that MFN double mutant spermatocytes cannot move to the pachytene stage at PD 24 (Varuzhanyan et al., 2019), and our data are once again consistent with their conclusions. By quantifying the number of spermatocytes at four different meiotic stages of prophase I, and combining these data with our apoptotic marker imaging analysis, we concluded that MFN2 deficiency leads to a failure of meiosis in spermatocytes, especially for the development from the zygotene to pachytene stages. In addition to the meiosis related markers, we also checked the chromosomal dynamics during meiosis. It is generally considered that when chromosomes search for a homologous partner in meiotic prophase, it is the telomere rather than the centromere that assembles the apparatus that binds to the nuclear envelope (Scherthan et al., 1996; Shibuya et al., 2014). The morphology of the chromosomes and telomeres were visualized by our SYCP3 and TRF1 dual-immunolabeling experiments, and our results demonstrated that the majority of telomeres were localized in the middle area of the nucleus in the MFN2 cKO group whereas they were localized at the equator in the MFN2 WT group. These data once again support our hypothesis that MFN2 deletion might disrupt the normal biological functions of the mitochondria, such as ATP synthesis and oxidative phosphorylation. The resulting lack of ATP might, in turn affect the normal events of meiosis, such as telomere associated chromosome dynamics. We also report that the normal chromosomal cross-over activity was affected in MFN2 cKO spermatocytes as there was almost no MLH1 expression at the mid-pachytene stage. Thus, we suggest that the MFN2 deficiency might be essential for the normal differentiation of spermatogonia and development of spermatocytes. When spermatocytes are deficient in MFN2, then they cannot continue to the pachytene stage and this leads to apoptosis, which ultimately results in the death of both the spermatocytes and spermatogonia in the seminiferous tubule, hence the empty appearance of this region in our histology sections. A similar phenotype has previously been reported in other studies (Varuzhanyan et al., 2019; Chen et al., 2020; Wang et al., 2021), but to our knowledge, our data are the first to show details of disrupted meiosis in MFN2 deficient spermatocytes.

Our mitochondrial distribution analysis indicated that mitochondria were aggregated in spermatocytes of MFN2 cKO mice. Previous studies have shown that in both MFN1 and MFN2 deficient mice, the mitochondria in embryonic fibroblasts exhibited a fragmented morphology, rather than the elongated morphology seen in the wild-type cells (Chen et al., 2003). This report described global knockout mouse models instead of the conditional KO model we generated. In addition, the embryonic fibroblasts that were examined in the previous report, are not representative of germ cells. Recently, this group also generated a conditional knock-out mouse model and investigate the distribution of mitochondria in spermatocytes via immunolabeling meiotic chromosome spreads (Varuzhanyan et al., 2019). They showed that in the pachytene/diplotene stages, WT spermatocytes contain a majority of elongated mitochondria whereas the spermatocytes from MFN double mutants exhibited mainly spherical mitochondria (Varuzhanyan et al., 2019). However, one tricky point in their study is that normally, cell membrane might be disrupted already and cytosol is possibly run out of cell in the meiotic spreads. To overcome this limitation, we used the cytospin technique to let the spermatocytes spread on the slides before the immunostaining process. This enabled us to collect the spermatocytes in a naturally round shape with an intact cell membrane and the entire cytosol. So, the mitochondria immunolabeled in each cell were closer to the real situation for quantitative analysis. In addition, the mtDNA copy number was found to be significantly increased in the MFN2 cKO spermatocytes, which was consistent with our immunolabeling data. Together, these data suggest that the total number of mitochondria was increased after MFN2 deletion. We also showed (by RT-qPCR) that the mitochondrial respiratory chain was disrupted in the MFN2 cKO cells, which might help to explain the low level of ATP synthesis and energy supply observed, and thus the various changes in critical cellular activities. In addition, we checked the level of ROS and mitochondrial membrane potential in the MFN2 cKO spermatocytes, and our data demonstrated that the cells had poor mitochondrial function. Our data thus provide evidence that MFN2 is required to maintain normal mitochondrial functions and MFN2 deficient spermatocytes might harbor mitochondria of poor quality, so they cannot go through meiosis successfully.

To compare the transcriptome status of the MFNs cKO and WT animals, we selected two developmental ages, PD 24 and PD 52, to conduct RNA-seq analysis. Pachytene cells were selected for analysis as they have previously been shown to be severely affected after deletion of MFN2, and this cell stage is large in size when compared with the other spermatocyte stages (La Salle et al., 2009). Therefore, we were able to collect this population of cells at a high purity. From our Venn analysis of both PD 24 and PD 52, we highlighted 135 genes that had significant changes in their expression levels. A KEGG analysis was then run on these genes based on the PD 24 pachytene spermatocyte data alone. This is because we believe that PD 52 is a relatively late stage for MFN2 cKO animals to be showing severe testicle structure abnormalities and spermatocyte death. Thus, we suggest that the analysis from PD 24 animals might provide more informative data. Interestingly, our KEGG analysis indicated that in the MFN2 cKO group, there were significant changes in cellular metabolism, especially for lipid related metabolism. In addition, our RNA-seq data highlighted different in PPAR signaling. PPARs belong to a family of nuclear receptors and they are transcriptional factors that can bind ligands to modulate cellular activities and regulate related gene expression (Brunmeir and Xu 2018). PPARs have previously been reported to coordinate lipid and glucose metabolism, and the abnormal expression of PPARs might lead to a perturbation of lipid metabolism, which is characteristic of various metabolic syndromes (Alatshan and Benkő 2021; Puengel et al., 2022). A variety of natural compounds, including fatty acids and their derivatives, have been found to bind and activate PPAR proteins (Monsalve et al., 2013; Brunmeir and Xu 2018). In this way, PPARs are recognized as being sensors of cellular metabolic states via their sensitivity to the intracellular levels of various metabolites (Maccallini et al., 2017; Mantovani et al., 2022). Interestingly, the different PPAR sub-families are suggested play distinct roles in the regulation of lipid metabolism (Alatshan and Benkő 2021). For example, PPAR α was reported to play a central role in the control of a lipid-lowering effect, which might help decrease the storage of lipids via regulating the expression of related enzymes (Han et al., 2017; Phua et al., 2018). Our data demonstrated the in MFN2-deficient spermatocytes, PPAR α was significantly increased, which might suggest a high level of lipid storage in MFN2 cKO testis tissue. PPAR *ß* is the most ubiquitously expressed member among PPAR family. It has been reported that the activation of PPAR *ß* might induce lipid catabolism through fatty acid oxidation and mitochondrial biogenesis, and it has also been reported to reduce inflammation via the inhibition of AKT, STAT3 and ERK1/2 signaling (Kapoor et al., 2010; Alatshan and Benkő 2021). According to these previous studies on PPAR β, we believe that it might play a similar role as PPAR α in MFN2 cKO animals, such that the up-regulation of PPAR α and PPAR *ß* is due to the increased storage of lipid in the MFN2 cKO testis. In contrast, PPAR γ has been suggested to play a different role in the regulation of lipid metabolism and it helps to increase lipid storage in the tissue and organs. It can also regulate the expression of mitochondrial proteins and members of the electron transport chain (Zhang and Chawla 2004). We know that in the MFN2 cKO group, mitochondrial functions and the electron transport chain were disrupted, and so this effect might be associated with the low expression of PPAR γ found in MFN2 deficient spermatocytes. From the perspective of lipid metabolism, our findings also indicate that there was only limited PPAR γ expression in the high lipid storage condition in the MFN2 cKO testis, this might indicate that the MFN2 cKO testis tissue do not need to store lipid anymore. Moreover, one previous publication reported the oxygen consumption level was low in the situation of low PPAR γ activity (de Vos et al., 2022). This links the mitochondrial function and PPAR γ activity, and our data here showed MFN2 cKO spermatocytes presented a declined mitochondrial function and decreased PPAR γ expression, which consists with this reported conclusion. Our RT-qPCR and western blotting data were consistent, which supports the accuracy of our conclusions. To further confirm whether the lipid metabolism was disrupted in the MFN2 cKO animals, we labeled testes cells with BODIPY to specifically label the lipid droplets. The images from the MFN2 WT control and MFN2 cKO groups revealed a high lipid storage level in the latter. Therefore, we suggest that MFN2 deficiency might lead to a disruption in lipid metabolism in the testicle cells. However, our current study does not have direct evidence to demonstrate the change in PPAR expression is the cause of lipid accumulation, further investigations are required to clarify how PPAR-mediated lipid metabolism plays a role in spermatogenesis and its relationship with lipid metabolism in spermatocyte development.

## Summary

Overall, our data showed that in MFN2 deficient spermatocytes, the function and distribution of mitochondria were disrupted. This affected meiosis and spermatogenesis, which resulted in germ cell apoptosis and abnormal morphology of the testis. We also demonstrated that the PPAR signaling pathway was significantly affected in the MFN2 deficient spermatocytes, and that there was increased lipid retention in the testicle cells of MFN2 deficient animals. Together, our data indicate that MFN2 is required for spermatogenesis and meiosis. In addition, we discovered a novel role of MFN2 in regulating PPAR pathway and lipid metabolism in testicle cells.

## Data Availability

The datasets presented in this study can be found in online repositories. The names of the repository/repositories and accession number(s) can be found below: https://www.ncbi.nlm.nih.gov/, GSE194377.
