# Supplementary material for: MFN2 Deficiency Impairs Mitochondrial Functions and PPAR Pathway During Spermatogenesis and Meiosis in Mice
Source: Front Cell Dev Biol. 2022 Apr 14;10:862506. doi: 10.3389/fcell.2022.862506 (PMC9046932; doi:10.3389/fcell.2022.862506)
Supplement: Supplementary file 1 [file DataSheet2.PDF]

Fig.1 MFN2 WT vs MFN2 cKO testes images

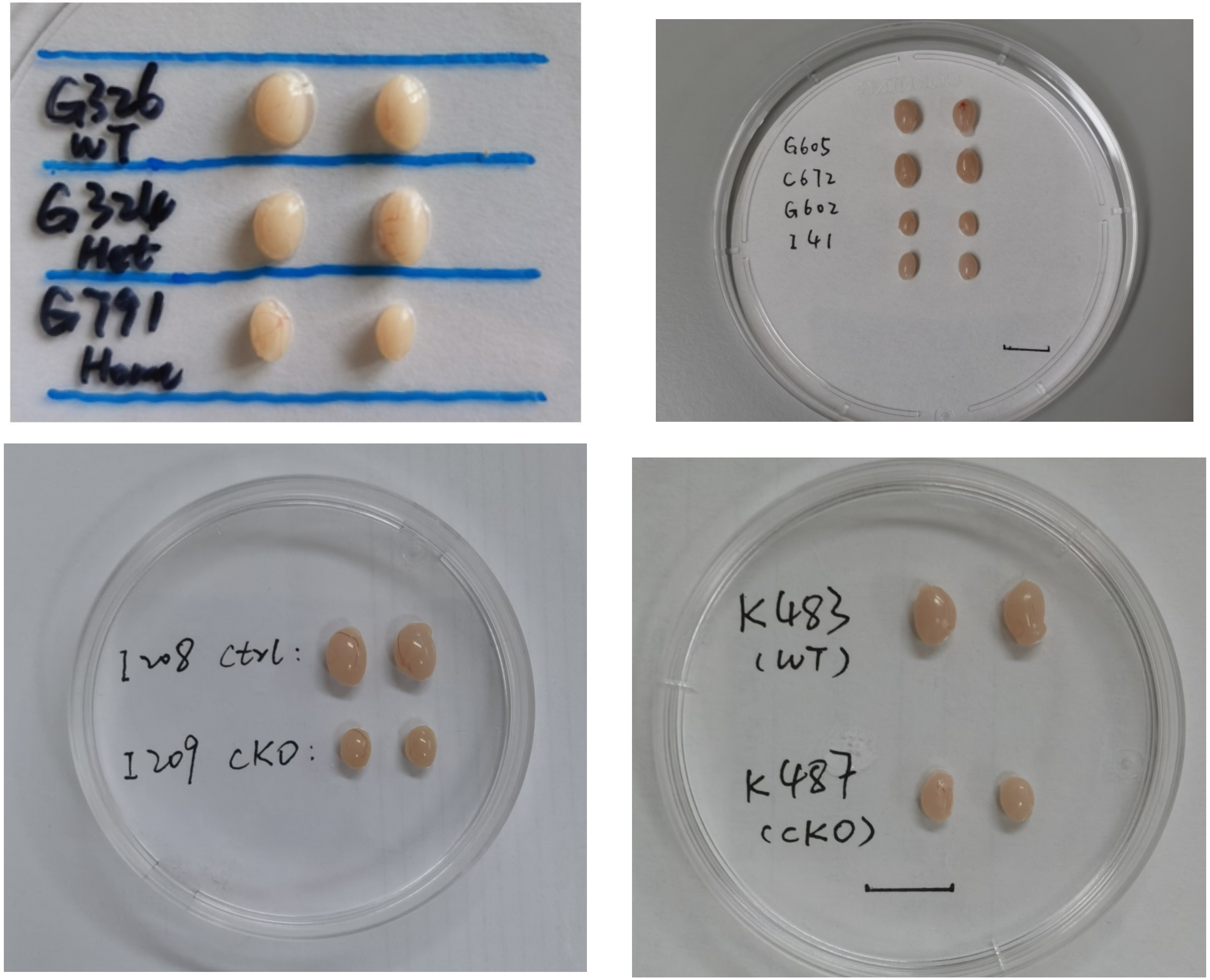

Fig.2

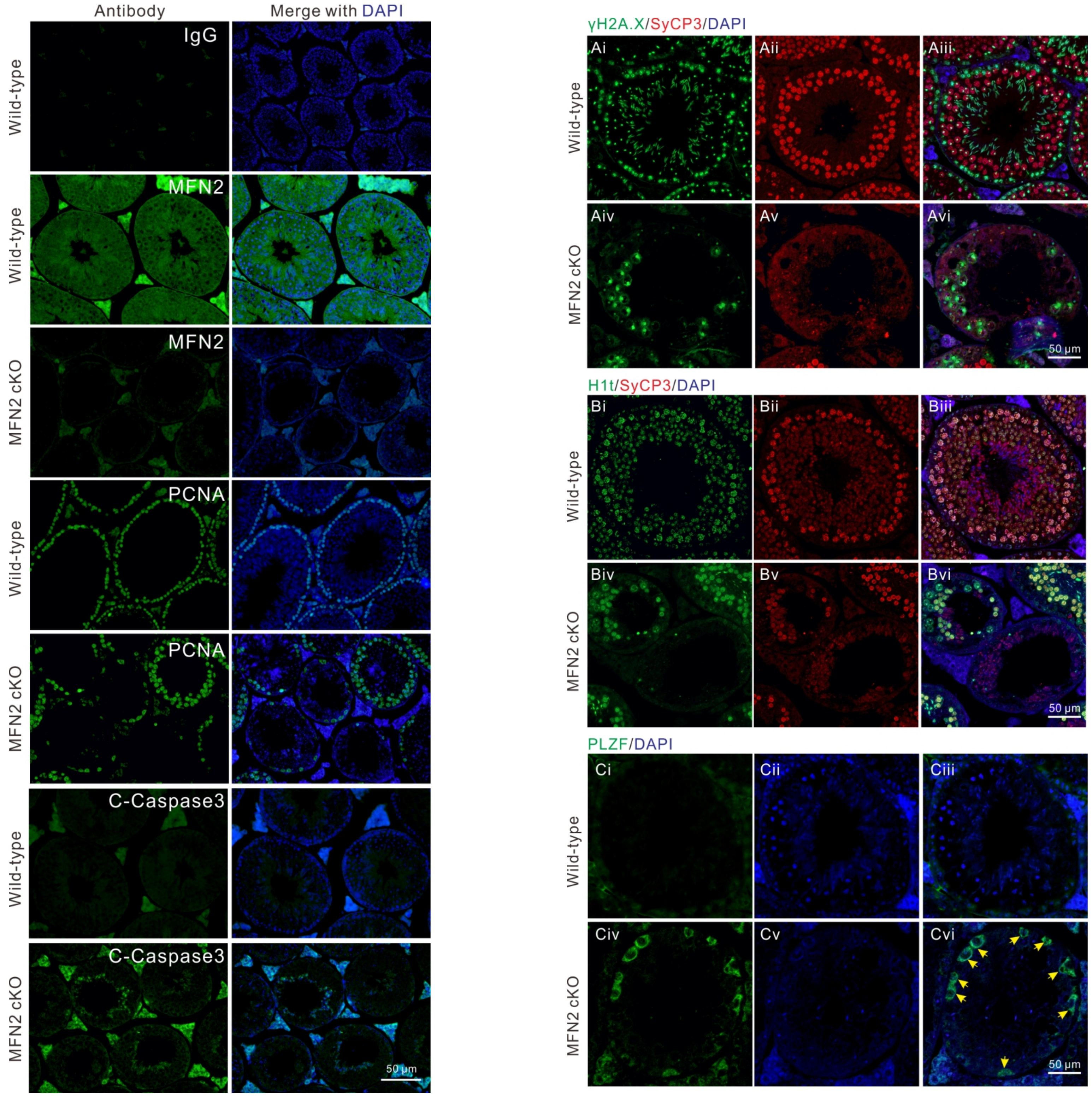
Antibody
Merge with DAPI
Wild-type
MFN2 cKO
IgG
MFN2
PCNA
C-Caspase3
50 µm
γH2A.X/SyCP3/DAPI
Ai
Aii
Aiii
Aiv
Av
Avi
H1t/SyCP3/DAPI
Bi
Bii
Biii
Biv
Bv
Bvi
PLZF/DAPI
Ci
Cii
Ciii
Civ
Cv
Cvi

Westerb Blot

For Fig.1 & 6

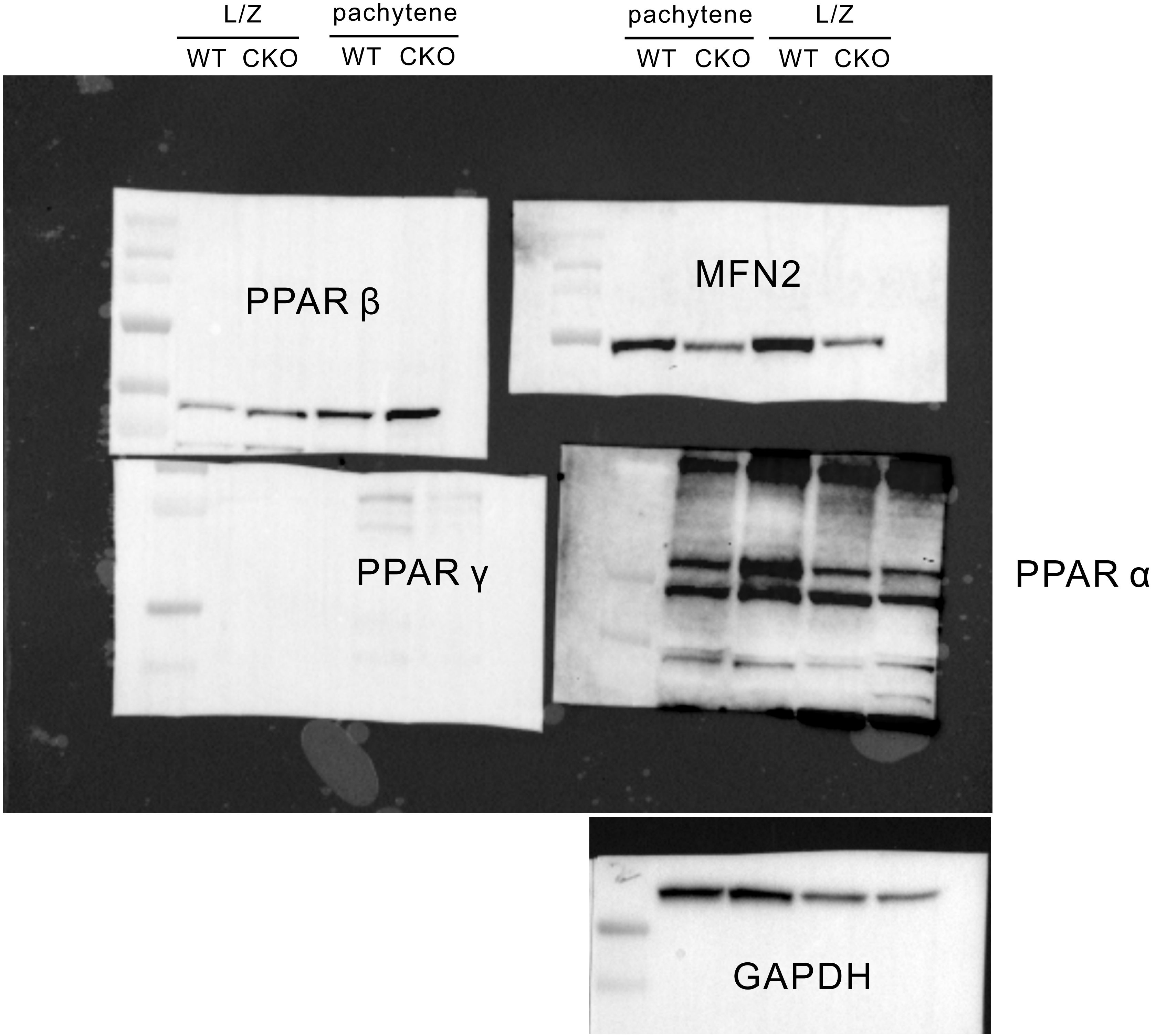

MFN2 cKO spermatocytes IF images

rH2A.X / Sycp3

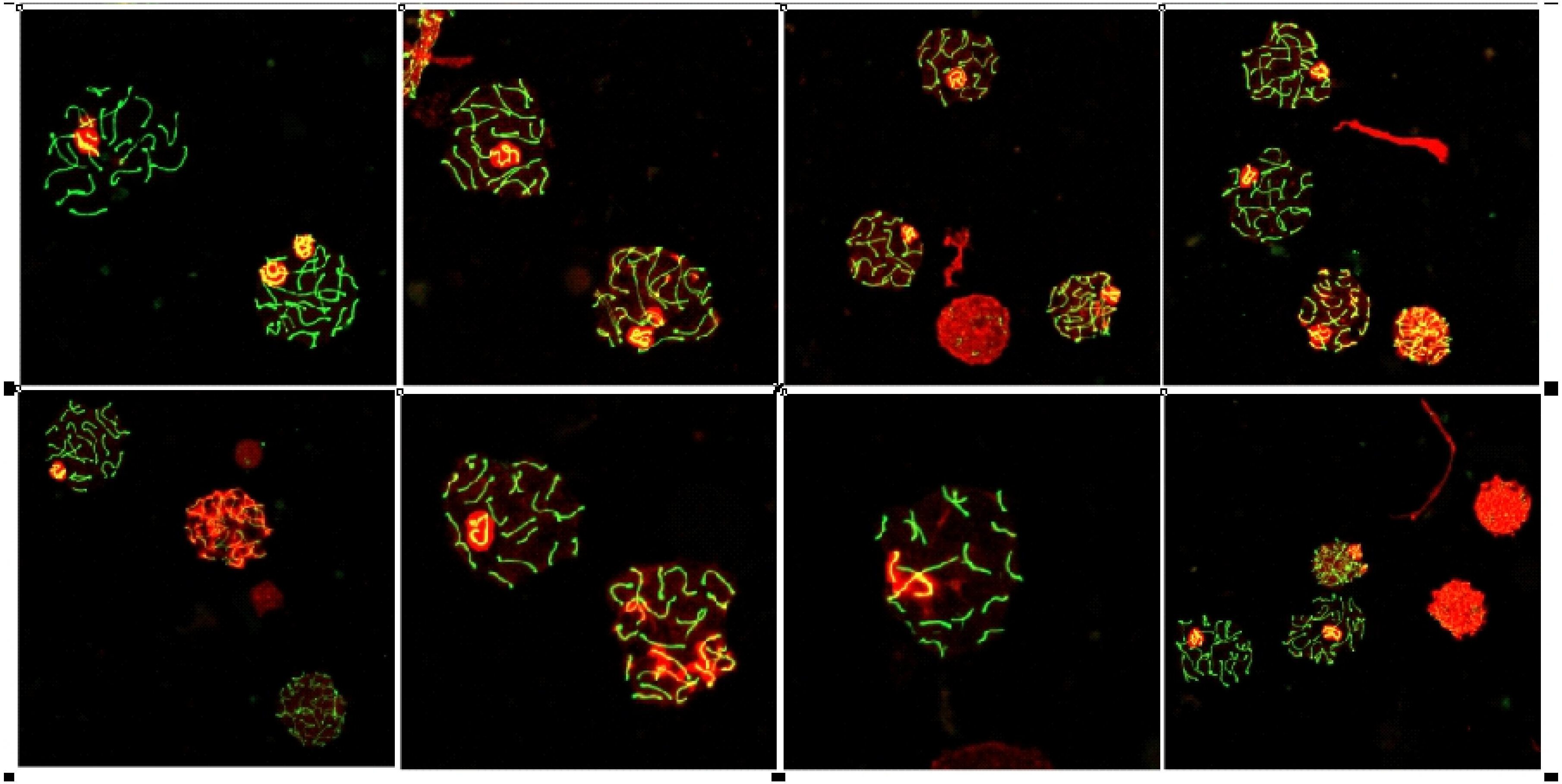

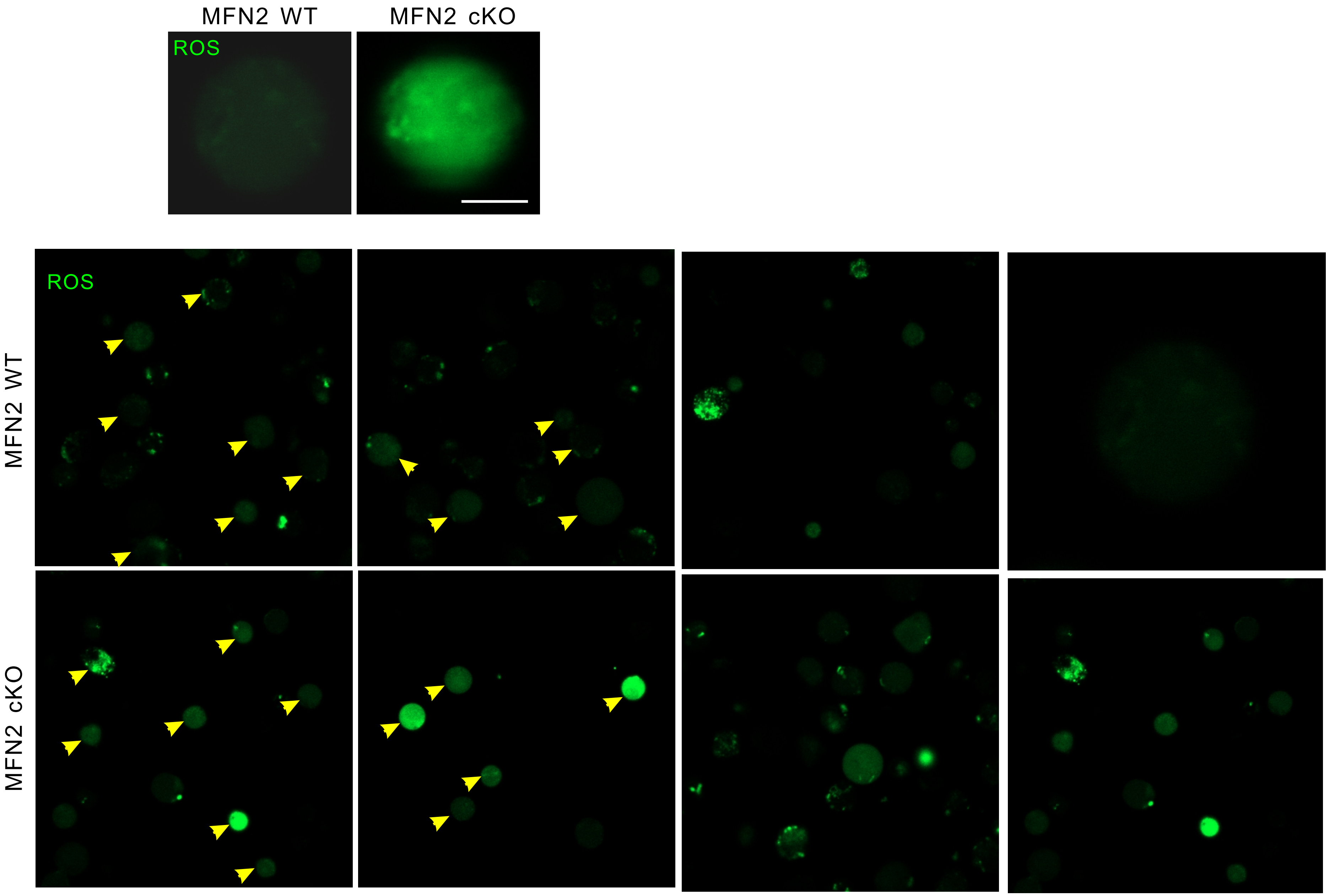
MFN2 WT
MFN2 cKO
ROS
ROS
MFN2 WT
MFN2 cKO

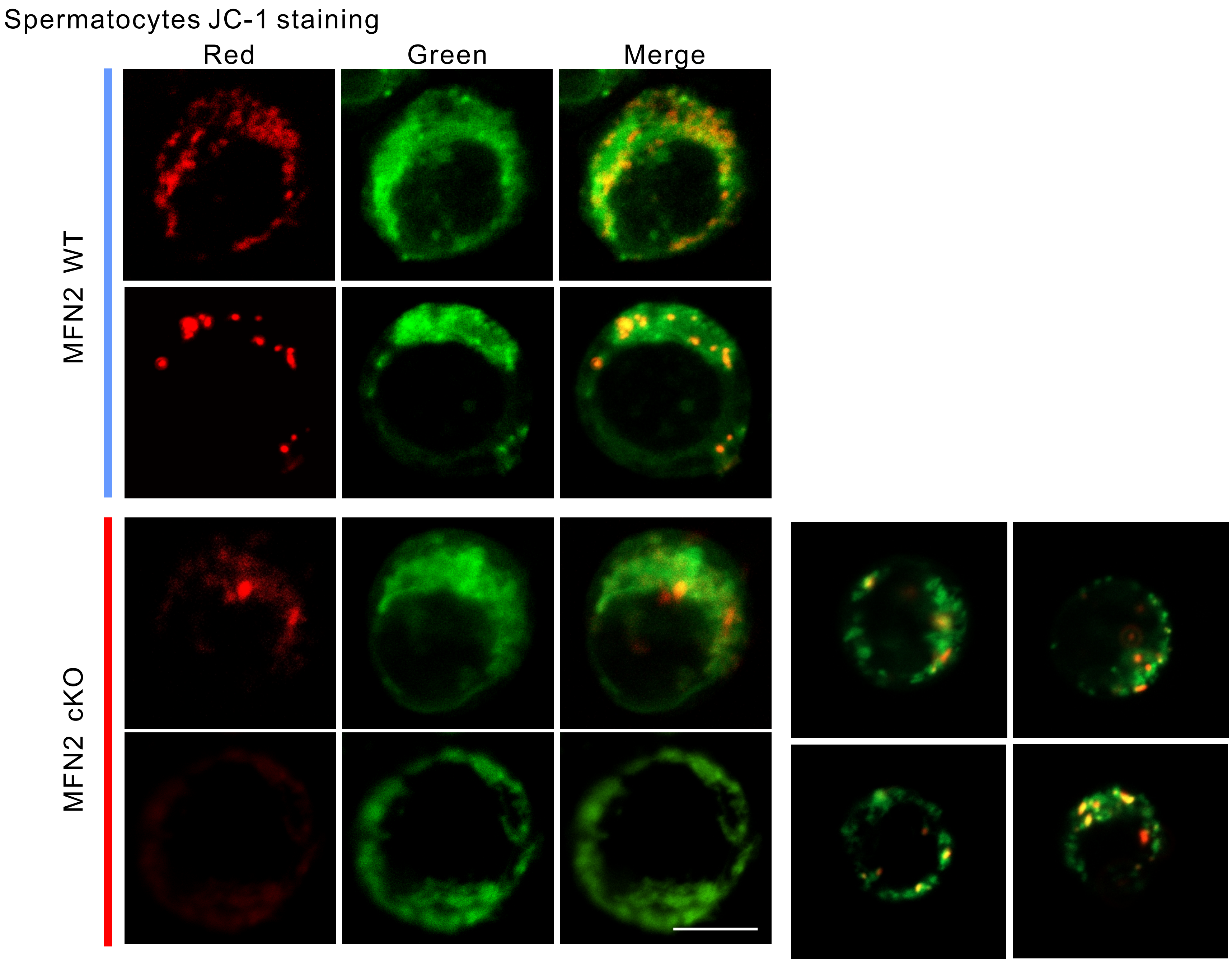
Spermatocytes JC-1 staining
Red
Green
Merge
MFN2 WT
MFN2 cKO

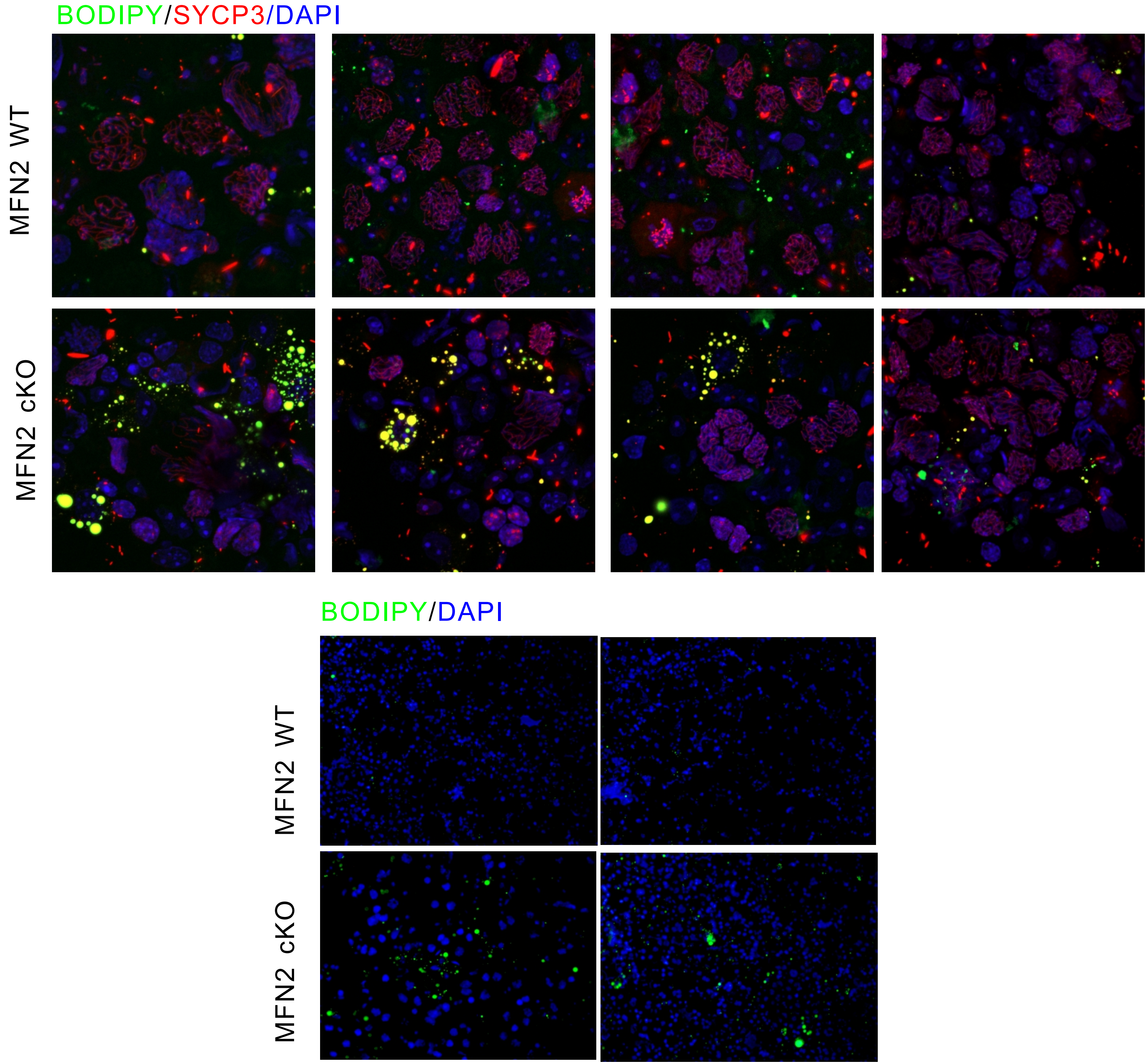
BODIPY/SYCP3/DAPI
MFN2 WT
MFN2 cKO
BODIPY/DAPI
MFN2 WT
MFN2 cKO

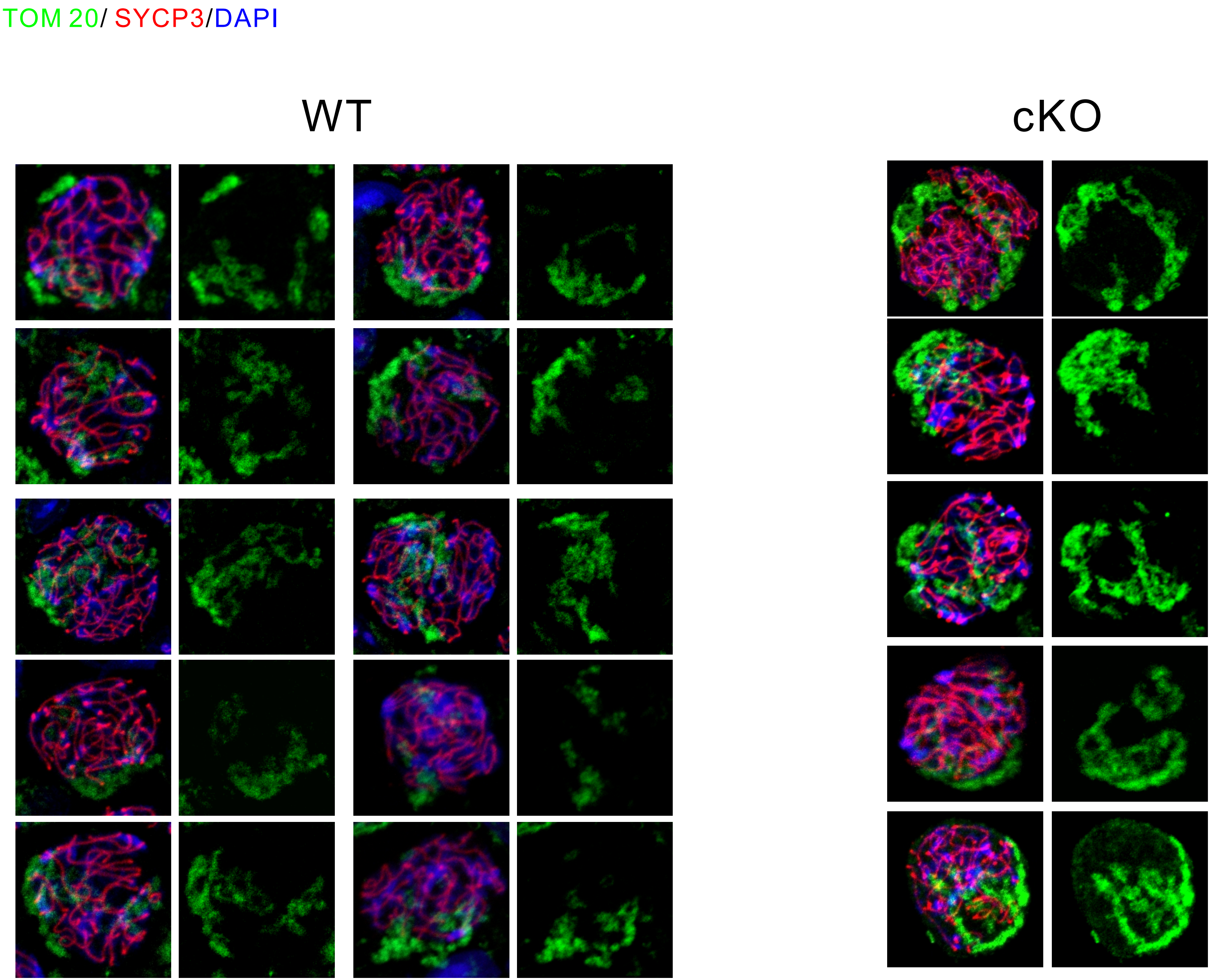
TOM 20/ SYCP3/DAPI
WT
cKO
