# Supplementary material for: MFN2 Deficiency Impairs Mitochondrial Functions and PPAR Pathway During Spermatogenesis and Meiosis in Mice
Source: Front Cell Dev Biol. 2022 Apr 14;10:862506. doi: 10.3389/fcell.2022.862506 (PMC9046932; doi:10.3389/fcell.2022.862506)
Supplement: Supplementary file 6 [file DataSheet3.PDF]

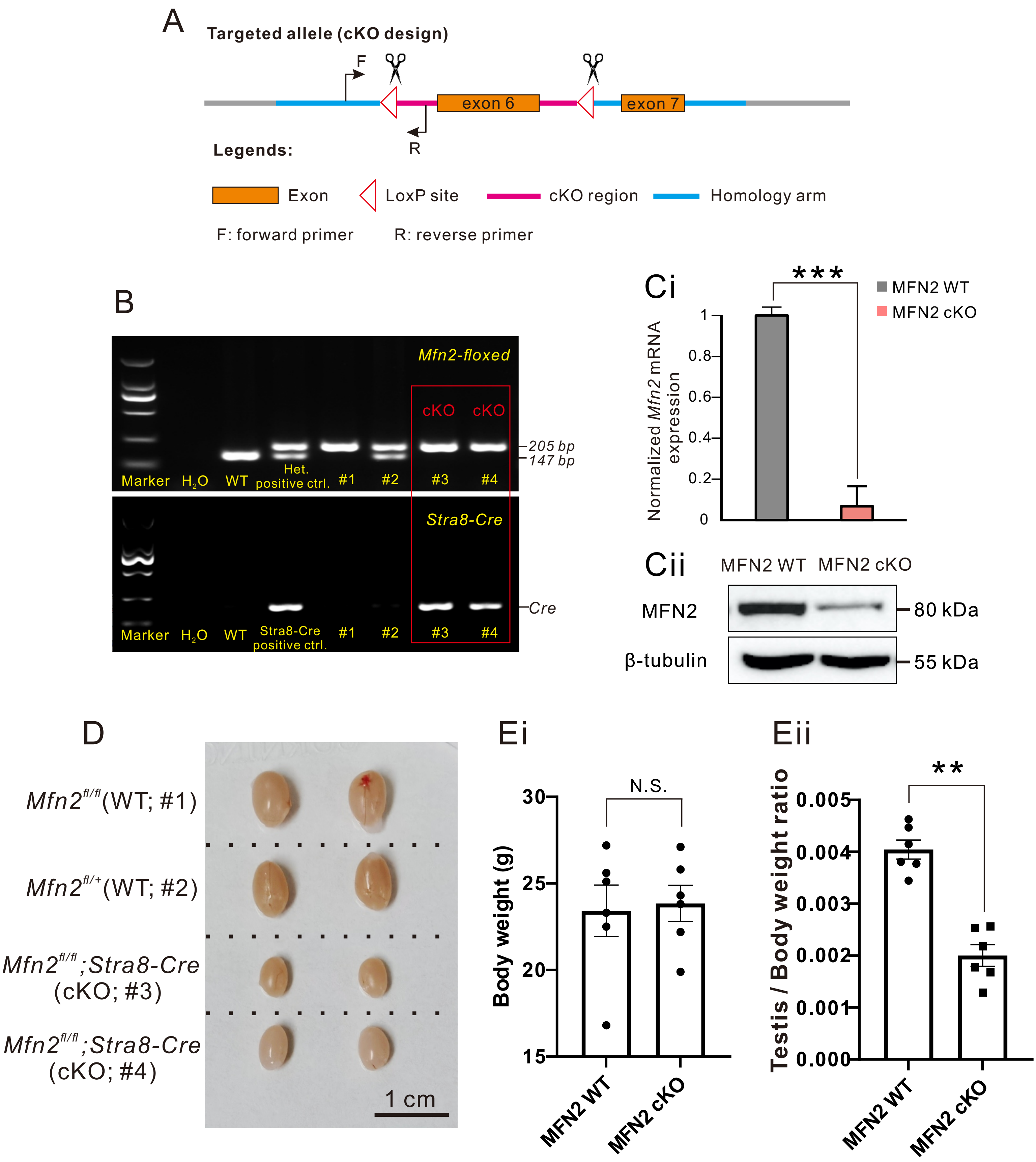
A
Targeted allele (cKO design)
F
exon 6
exon 7
R
Legends:
Exon
LoxP site
cKO region
Homology arm
F: forward primer
R: reverse primer
B
Mfn2-floxed
cKO cKO
205 bp
147 bp
Marker H2O WT Het. positive ctrl. #1 #2 #3 #4
Stra8-Cre
Cre
Marker H2O WT Stra8-Cre positive ctrl. #1 #2 #3 #4
Ci
***
MFN2 WT
MFN2 cKO
Normalized Mfn2 mRNA expression
1
0.8
0.6
0.4
0.2
0
Cii
MFN2 WT MFN2 cKO
MFN2
80 kDa
β-tubulin
55 kDa
D
Mfn2fl/fl(WT; #1)
Mfn2fl/+(WT; #2)
Mfn2fl/fl;Stra8-Cre (cKO; #3)
Mfn2fl/fl;Stra8-Cre (cKO; #4)
1 cm
Ei
N.S.
Body weight (g)
30
25
20
15
MFN2 WT
MFN2 cKO
Eii
**
Testis / Body weight ratio
0.005
0.004
0.003
0.002
0.001
0.000
MFN2 WT
MFN2 cKO

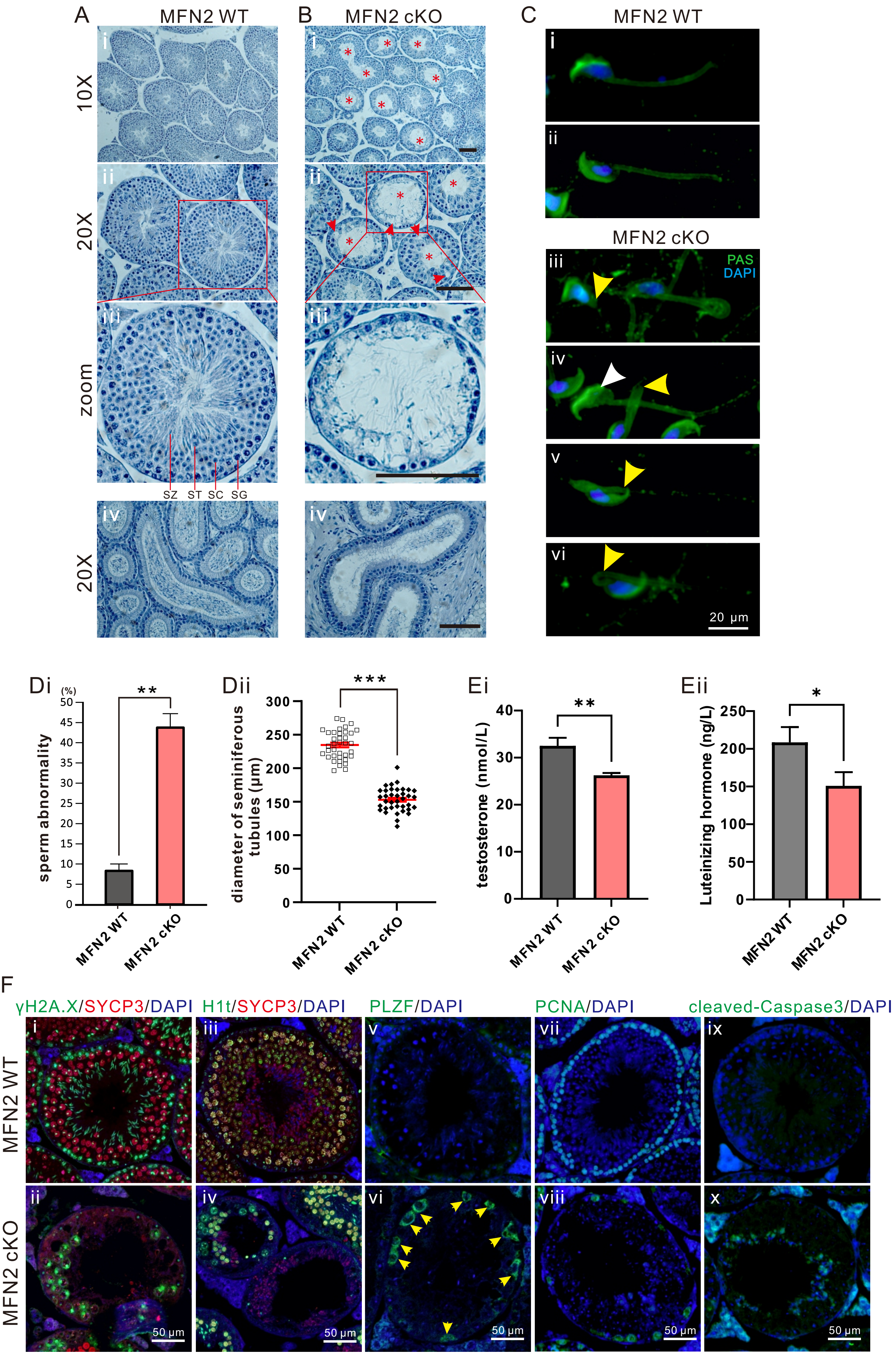

A
MFN2 WT
B
MFN2 cKO
C
MFN2 WT
10X
20X
zoom
20X
i
ii
iii
iv
SZ ST SC SG
MFN2 cKO
PAS
DAPI
v
vi
20 μm
Di
(%)
sperm abnormality
**
MFN2 WT
MFN2 cKO
Dii
diameter of seminiferous tubules (μm)
***
Ei
testosterone (nmol/L)
**
Eii
Luteinizing hormone (ng/L)
*
F
γH2A.X/SYCP3/DAPI
H1t/SYCP3/DAPI
PLZF/DAPI
PCNA/DAPI
cleaved-Caspase3/DAPI
vii
viii
ix
x
50 μm

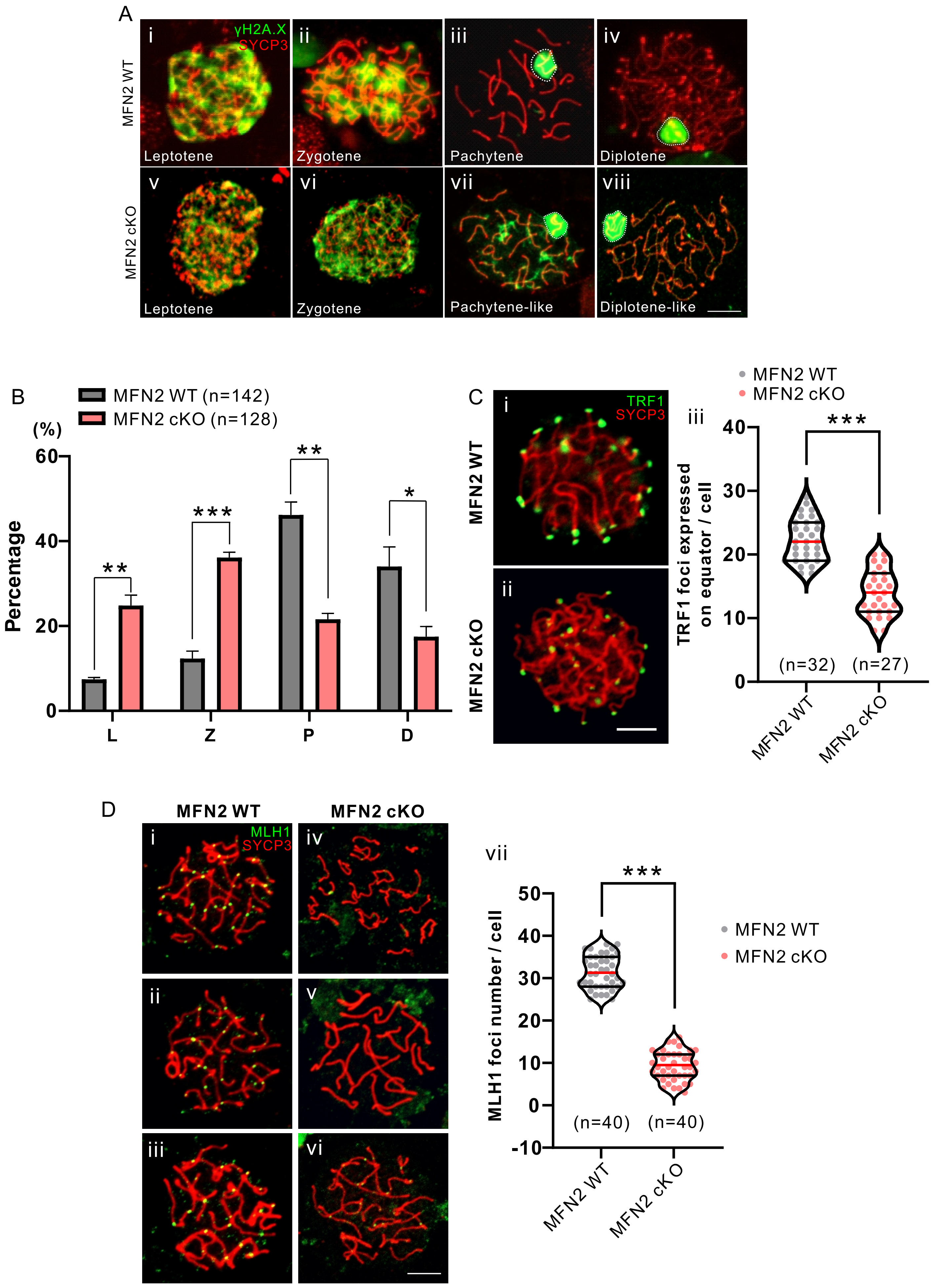

A
γH2A.X
SYCP3
MFN2 WT
MFN2 cKO
i
ii
iii
iv
v
vi
vii
viii
Leptotene
Zygotene
Pachytene
Diplotene
Pachytene-like
Diplotene-like
B
MFN2 WT (n=142)
MFN2 cKO (n=128)
(%)
Percentage
60
40
20
0
L
Z
P
D
C
TRF1
SYCP3
TRF1 foci expressed on equator / cell
40
30
20
10
0
(n=32)
(n=27)
D
MLH1
SYCP3
MLH1 foci number / cell
50
40
30
20
10
0
-10
(n=40)
(n=40)
MFN2 WT
MFN2 cKO

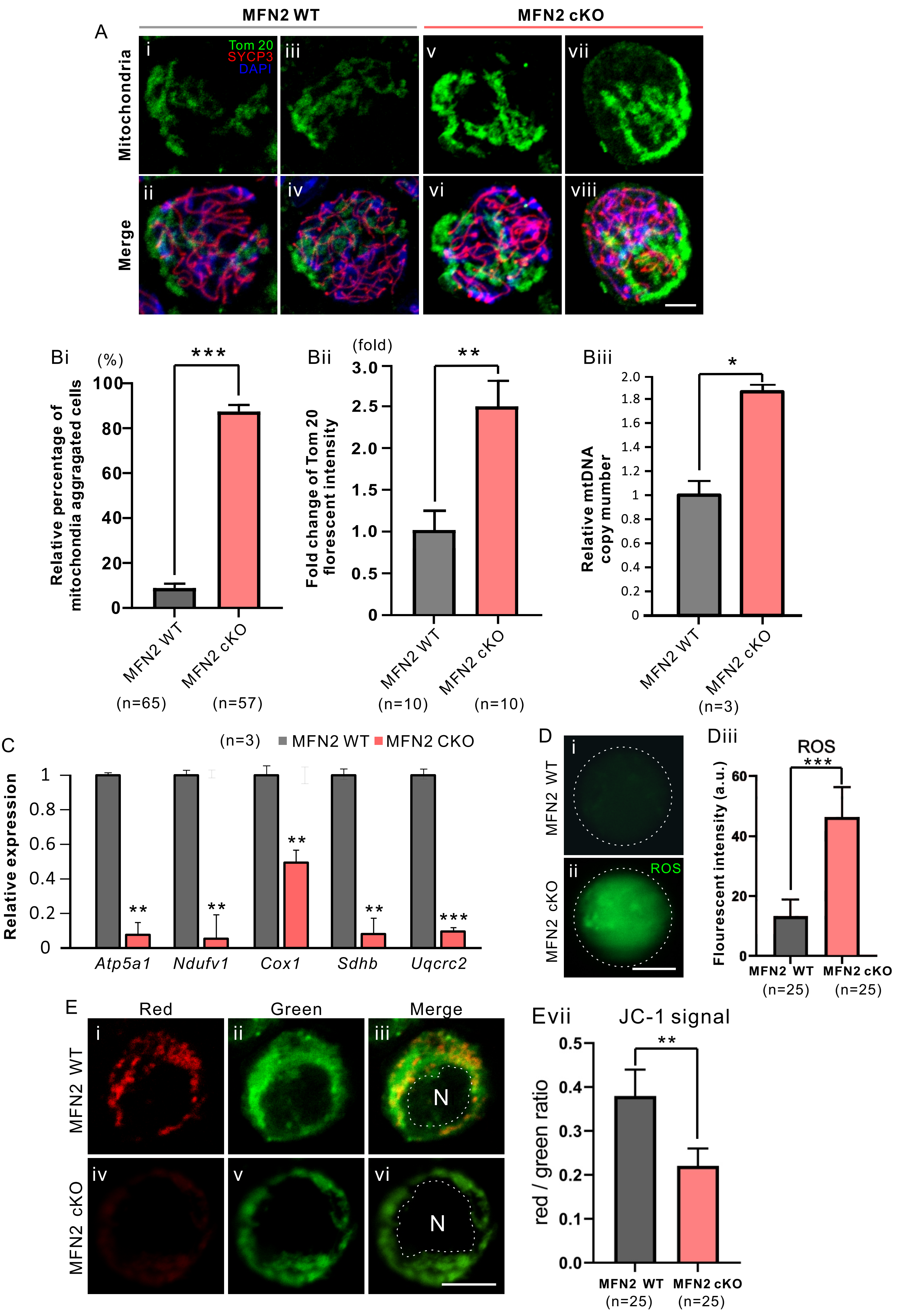

A
MFN2 WT
MFN2 cKO
Tom 20
SYCP3
DAPI
Mitochondria
Merge
i
ii
iii
iv
v
vi
vii
viii
Bi
(%)
Relative percentage of mitochondria aggragated cells
MFN2 WT
MFN2 cKO
(n=65)
(n=57)
Bii
(fold)
Fold change of Tom 20 florescent intensity
MFN2 WT
MFN2 cKO
(n=10)
(n=10)
Biii
Relative mtDNA copy mumber
MFN2 WT
MFN2 cKO
(n=3)
C
(n=3)
MFN2 WT
MFN2 CKO
Relative expression
Atp5a1
Ndufv1
Cox1
Sdhb
Uqcrc2
D
MFN2 WT
MFN2 cKO
ROS
Diii
ROS
Flourescent intensity (a.u.)
MFN2 WT
MFN2 cKO
(n=25)
(n=25)
E
Red
Green
Merge
MFN2 WT
MFN2 cKO
N
Evii
JC-1 signal
red / green ratio
MFN2 WT
MFN2 cKO
(n=25)
(n=25)

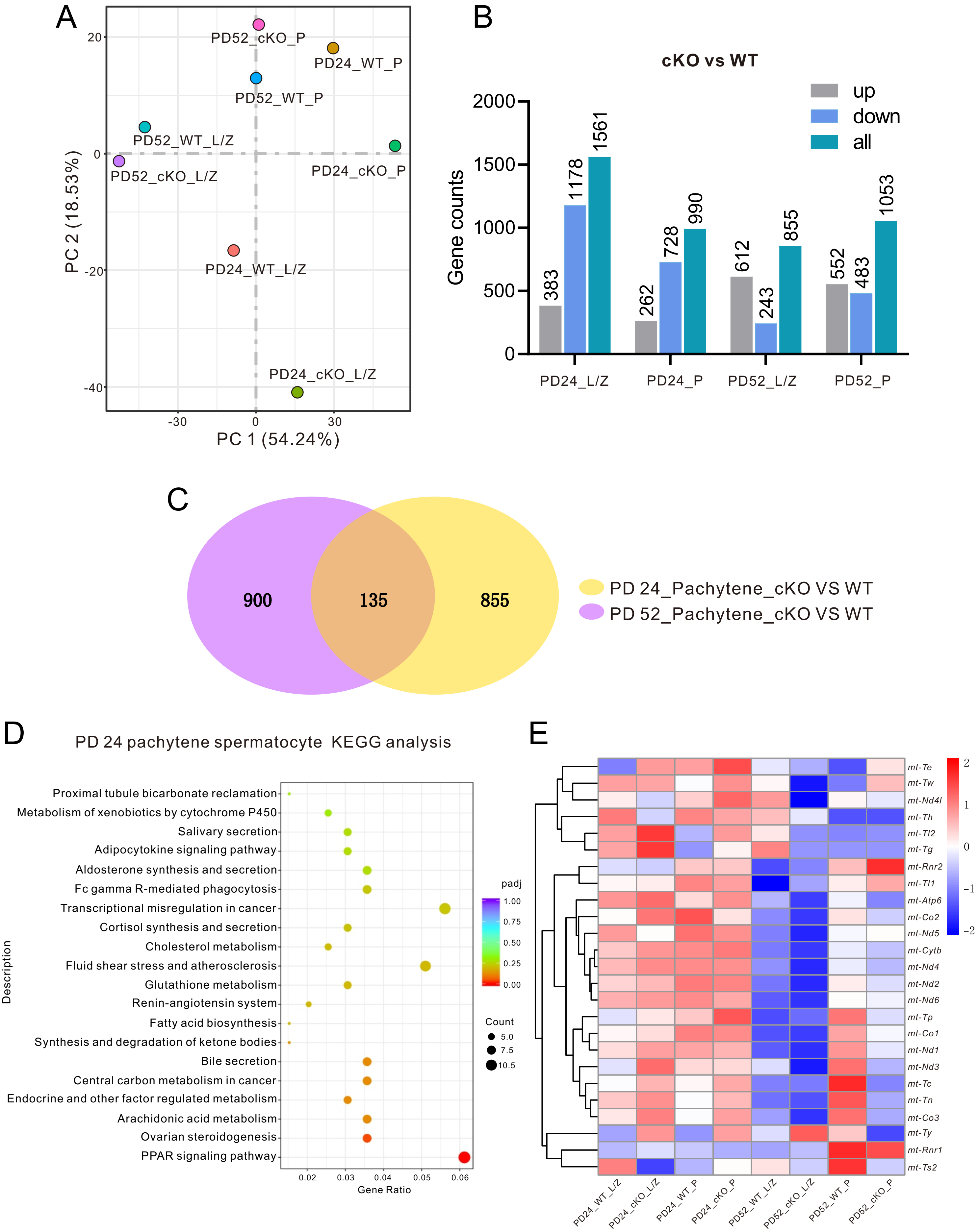
A
PC 1 (54.24%)
PC 2 (18.53%)
PD52_cKO_P
PD24_WT_P
PD52_WT_P
PD52_WT_L/Z
PD24_cKO_P
PD52_cKO_L/Z
PD24_WT_L/Z
PD24_cKO_L/Z
20
0
-20
-40
-30
0
30
B
cKO vs WT
Gene counts
up
down
all
2000
1500
1000
500
0
383
1178
1561
262
728
990
612
243
855
552
483
1053
PD24_L/Z
PD24_P
PD52_L/Z
PD52_P
C
900
135
855
PD 24_Pachytene_cKO VS WT
PD 52_Pachytene_cKO VS WT
D
PD 24 pachytene spermatocyte KEGG analysis
Description
Proximal tubule bicarbonate reclamation
Metabolism of xenobiotics by cytochrome P450
Salivary secretion
Adipocytokine signaling pathway
Aldosterone synthesis and secretion
Fc gamma R-mediated phagocytosis
Transcriptional misregulation in cancer
Cortisol synthesis and secretion
Cholesterol metabolism
Fluid shear stress and atherosclerosis
Glutathione metabolism
Renin-angiotensin system
Fatty acid biosynthesis
Synthesis and degradation of ketone bodies
Bile secretion
Central carbon metabolism in cancer
Endocrine and other factor regulated metabolism
Arachidonic acid metabolism
Ovarian steroidogenesis
PPAR signaling pathway
0.02
0.03
0.04
0.05
0.06
Gene Ratio
padj
1.00
0.75
0.50
0.25
0.00
Count
5.0
7.5
10.5
E
mt-Te
mt-Tw
mt-Nd4l
mt-Th
mt-Tl2
mt-Tg
mt-Rnr2
mt-Tl1
mt-Atp6
mt-Co2
mt-Nd5
mt-Cytb
mt-Nd4
mt-Nd2
mt-Nd6
mt-Tp
mt-Co1
mt-Nd1
mt-Nd3
mt-Tc
mt-Tn
mt-Co3
mt-Ty
mt-Rnr1
mt-Ts2
2
1
0
-1
-2
PD24_WT_L/Z
PD24_cKO_L/Z
PD24_WT_P
PD24_cKO_P
PD52_WT_L/Z
PD52_cKO_L/Z
PD52_WT_P
PD52_cKO_P

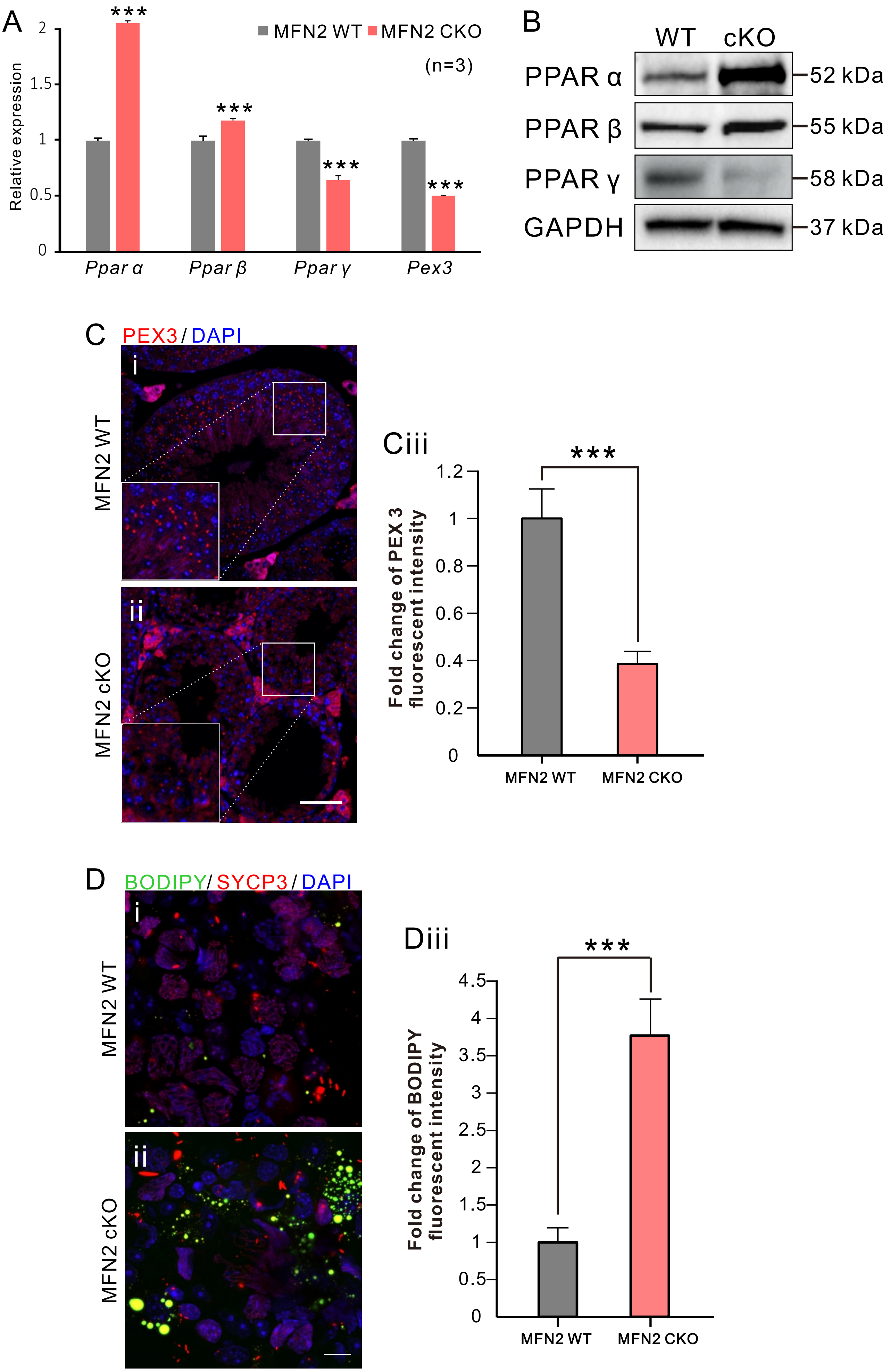
A
MFN2 WT
MFN2 CKO
(n=3)
Relative expression
***
Ppar α
Ppar β
Ppar γ
Pex3
B
WT
cKO
PPAR α
PPAR β
PPAR γ
GAPDH
52 kDa
55 kDa
58 kDa
37 kDa
C
PEX3 / DAPI
i
ii
MFN2 WT
MFN2 cKO
Ciii
Fold change of PEX 3 fluorescent intensity
MFN2 WT
MFN2 CKO
D
BODIPY / SYCP3 / DAPI
i
ii
MFN2 WT
MFN2 cKO
Diii
Fold change of BODIPY fluorescent intensity
MFN2 WT
MFN2 CKO

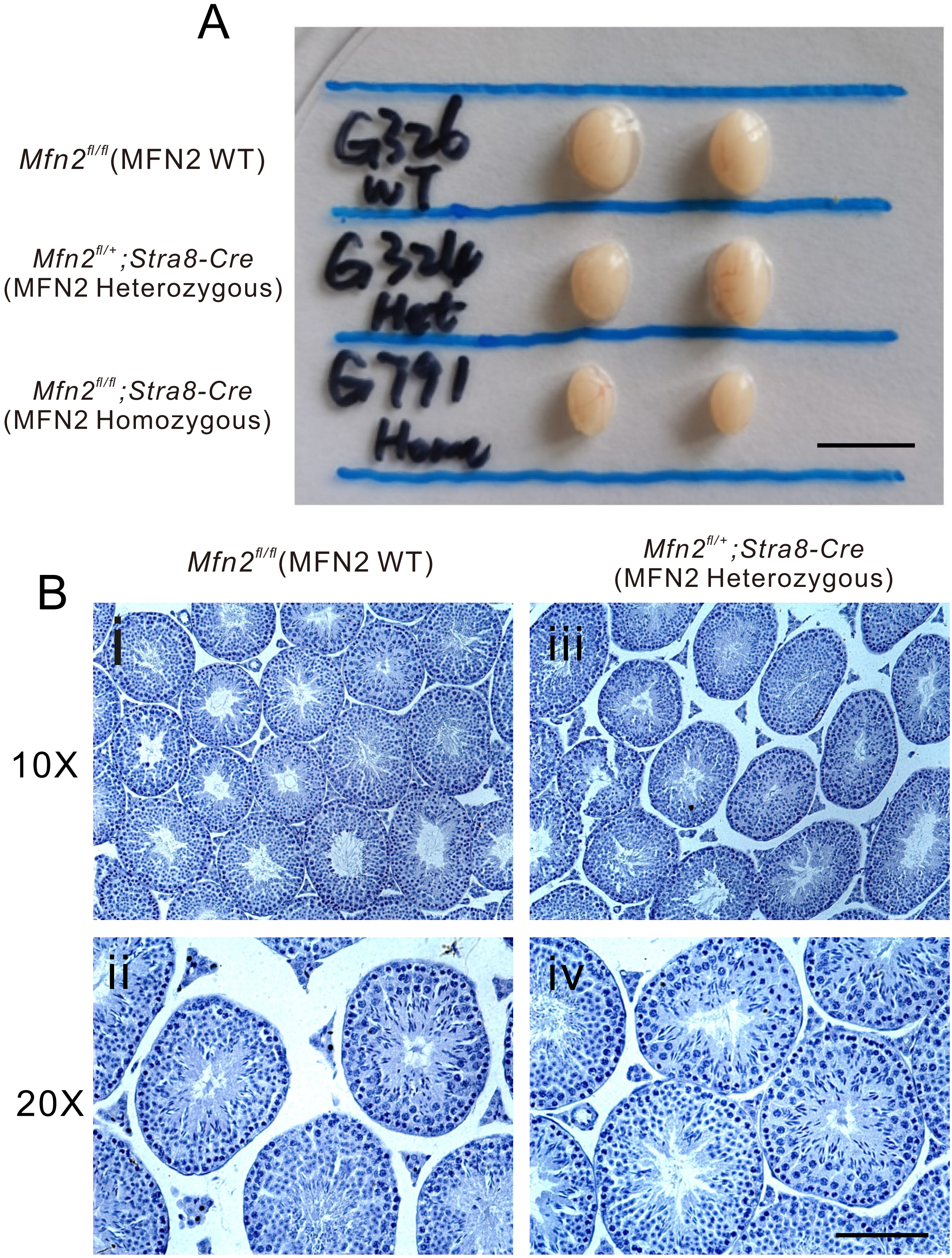

Fig. S1 (A) Representative examples of the testes excised from MFN2 WT (G326), MFN2 heterozygous (G324) and MFN2 homozygous cKO (G791) mice, which were collected on postnatal day (PD) 56. The scale bar is 1 cm. (B) Testes sections acquired at postnatal day (PD) 56 from (Bi-Bii) MFN2 WT and (Biii-Biv) MFN2 heterozygous mice, were stained with hematoxylin before imaged under a light microscope at 10X and 20X magnifications. Scale bar is 100 μm.
